# Transmembrane serine protease 6, a novel target for inhibition of neuronal tumor growth

**DOI:** 10.1038/s41419-024-06442-x

**Published:** 2024-01-13

**Authors:** Yong Zuo, Jiawei Bai, Huiyuan Bai, Siyu Tian, Hongtao Sun, Zhenhua Shi, Peng Yu, Guofen Gao, Yuan Li, Yan-Zhong Chang

**Affiliations:** Laboratory of Molecular Iron Metabolism, Key Laboratory of Molecular and Cellular Biology of Ministry of Education, Hebei Key Laboratory of Animal Physiology, Biochemistry and Molecular Biology, Hebei Collaborative Innovation Center for Eco-Environment, Hebei Research Center of the Basic Discipline of Cell Biology, College of Life Sciences, Hebei Normal University, Shijiazhuang, 050024 China

**Keywords:** Tumour-suppressor proteins, Apoptosis, Phosphoinositol signalling

## Abstract

Transmembrane serine protease 6 (Tmprss6) has been correlated with the occurrence and progression of tumors, but any specific molecular mechanism linking the enzyme to oncogenesis has remained elusive thus far. In the present study, we found that Tmprss6 markedly inhibited mouse neuroblastoma N2a (neuro-2a) cell proliferation and tumor growth in nude mice. Tmprss6 inhibits Smad1/5/8 phosphorylation by cleaving the bone morphogenetic protein (BMP) co-receptor, hemojuvelin (HJV). Ordinarily, phosphorylated Smad1/5/8 binds to Smad4 for nuclear translocation, which stimulates the expression of hepcidin, ultimately decreasing the export of iron through ferroportin 1 (FPN1). The decrease in cellular iron levels in neuro-2a cells with elevated Tmprss6 expression limited the availability of the metal forribo nucleotide reductase activity, thereby arresting the cell cycle prior to S phase. Interestingly, Smad4 promoted nuclear translocation of activating transcription factor 3 (ATF3) to activate the p38 mitogen-activated protein kinases signaling pathway by binding to ATF3, inducing apoptosis of neuro-2a cells and inhibiting tumor growth. Disruption of ATF3 expression significantly decreased apoptosis in Tmprss6 overexpressed neuro-2a cells. Our study describes a mechanism whereby Tmprss6 regulates the cell cycle and apoptosis. Thus, we propose Tmprss6 as a candidate target for inhibiting neuronal tumor growth.

## Introduction

A tumor of the sympathetic nervous system, neuroblastoma (NB) is one of the most common tumors in children [1, 2]. The incidence of NB is estimated at 1.2 cases per 100,000 people, accounting for about 15% of all cancer deaths in children [3–6]. The survival rate of low- and medium-risk patients is close to 100%, but the 5-year survival rate of high-risk NB patients is lower than 50% [7–9]. Understanding the mechanism of NB is the key to its treatment; however, despite many advances over the past three decades, the elusive mechanism of NB carcinogenesis has been a difficult challenge for clinical and basic researchers [10].

Iron (Fe) is essential to cell proliferation [11]; tumor cells require more iron than normal cells in order to support the rapid growth of the neoplasm [12]. Ribonucleotide reductase (RNR) catalyzes the rate-limiting step in deoxynucleotide synthesis. The enzyme catalyzes the de novo synthesis of deoxynucleotide triphosphates (dNTPs), generating 2-deoxynucleotide through reduction of carbon atom 2 of 5-phosphate ribose; the formed deoxynucleotide is then used for DNA synthesis [13]. The activation of RNR is dependent on Fe, since the enzyme complex’ R2 subunit contains a tyrosyl radical that is stabilized by Fe. In addition, DNA polymerases, primers, and helicases that play important roles in DNA replication are dependent on Fe^2+^ or iron-sulfur (Fe-S) clusters [12, 14]. Thus, Fe may be considered a cofactor for tumor cell proliferation.

Cancer growth can be seen as an imbalance between cell gain and cell loss, with mutated tumor cells multiplying faster than they die [15]. Apoptosis is a key physiological mechanism that limits cell population expansion, either to maintain tissue homeostasis or to eliminate potentially harmful cells, such as those with DNA damage [16]. As a CREB/ATF family member, ATF3 is frequently up-regulated by a wide variety of intra- and extracellular stressors [17]. ATF3 plays a key role in regulating cell behavior by homo- or hetero-dimerizing with ATF members, activating or inhibiting downstream genes [18]. Several studies have shown that ATF3 plays an important role in apoptosis by regulating downstream signaling pathways, such as ERK1/2, JNK, P38, and NF-κB [19, 20].

The type II transmembrane serine protease (TTSP) family is a class of proteolytic enzymes that are fixed to the cell membrane through the transmembrane region of the amino terminus [21]. The location of these proteins on the surface of cells puts them in a unique position to mediate signal transduction between cells and their surroundings, endowing this family of enzymes with important roles in many biological processes in mammals [22]. There are 17 TTSP members in humans. Tmprss6 is one of these and plays a key role in iron homeostasis by modulating hepcidin, a hepatic peptide hormone that binds to and downregulates ferroportin 1 (FPN1), the only known cellular iron transporter. Interestingly, Tmprss6 expression has been reported in breast and prostate cancers [23, 24]; however, little is known about the molecular function of Tmprss6 in cancer. Here, we show that overexpression of Tmprss6 significantly inhibited the proliferation of neuro-2a cells, stimulating significant cell death. Our results identify Tmprss6 as a new target for inhibiting the growth of neuronal tumors.

## Results

### Overexpression of Tmprss6 in neuro-2a cells

**Fig. 1:**
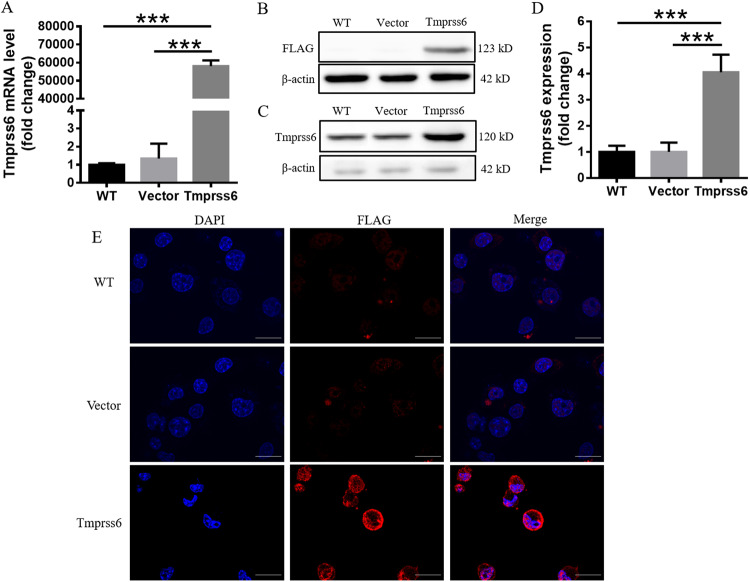
Overexpression of Tmprss6 in neuro-2a cells. **A** qRT-PCR detection of Tmprss6 mRNA expression in neuro-2a cells. Western blot detection of FLAG **B** and Tmprss6 **C** expression in neuro-2a cells. A representative blot image for each protein and the respective β-actin levels are shown. Quantification of Tmprss6 **D** expression in neuro-2a cells. Each sample’s relative expression level was calculated by normalizing its specific band to its corresponding β-actin or Histone3 band, and then comparing the mean ratio to that of the control group to get the relative fold change. **E** Immunofluorescence was used to detect the location of FLAG tags in neuro-2a cells (Scale bar = 20 μm). Data are expressed as the mean ± SD, *n* = 4. ∗∗∗*p* < 0.001.

To explore the relationship between Tmprss6 and NB, we manipulated the levels of Tmprss6 in the NB cell line, neuro-2a [25]. We confirmed increases in Tmprss6 and FLAG expression in the cells by qRT-PCR and western blot analysis, respectively (Fig. 1A–D), and the distribution of the overexpressed protein on the neuro-2a cell membrane by immunofluorescence staining (Fig. 1E). These results indicate successful overexpression of Tmprss6 in neuro-2a cells.

### Tmprss6 overexpression inhibits the Bmp-Smad signaling pathway and regulates the expression of hepcidin by cleaved HJV

**Fig. 2:**
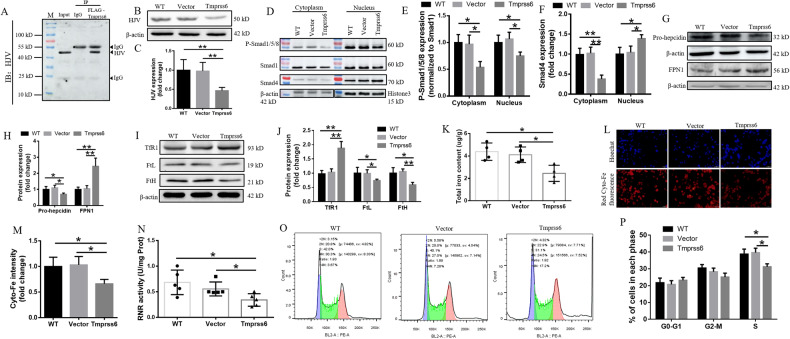
Effect of Tmprss6 overexpression on the cell cycle, iron content, and Bmp-Smad signaling pathway. **A** The interaction between FLAG (Tmprss6) and HJV was detected by immunoprecipitation. **B** Western blot analysis of HJV expression in neuro-2a cells (*n* = 4). **C** Quantification of HJV expression from the experiment shown in **B**. **D** The expression of P-Smad1/5/8, Smad1 and Smad4 proteins in the cytoplasm and nucleus were detected by western blot analysis (*n* = 4). Quantification of P-Smad1/5/8 / Smad1 **E** and Smad4 **F** expression from the experiment shown in **D**. **G** Western blot analysis of pro-hepcidin and FPN1 levels in neuro-2a cells (*n* = 4). **H** Quantification of pro-hepcidin and FPN1 expression from the experiment shown in **G**. **I** Western blot analysis of FtH, FtL, and TfR1 levels in neuro-2a cells (*n* = 4). **J** Quantification of FtH, FtL, and TfR1 levels from the experiment shown in **I**. Each sample’s relative expression level was calculated as described for Fig. 1D. **K** Total intracellular iron content was determined by ICP-MS (*n* = 4). **L** FerroOrange probe was used to detect intracellular Fe^2+^ content (Scale bar = 100 μm). **M** Quantitative evaluation of Fe^2+^ staining. **N** RNR activity was tested using a kit from Mlbio (*n* = 5). **O** The cell cycle was assessed by flow cytometry. **P** The percentage of cells in the G0/G1, S, and G2/M phases. Data are presented as the mean ± SD. ∗*p* < 0.05, ∗∗*p* < 0.01.

To identify the role of Tmprss6 in neuro-2a cells, we evaluated the levels of HJV, a substrate of Tmprss6 [26]. As expected, Tmprss6 overexpression (Tmprss6 group) decreased the levels of HJV via cleavage of the proteins compared with the Vector and WT groups (Fig. 2A–C). Since HJV is a co-receptor of Bmp [27], a decrease in HJV levels inhibits cytosolic Smad1/5/8 phosphorylation (P-Smad1/5/8) and significantly reduces the levels of P-Smad1/5/8 in the nucleus (Fig. 2D, E). Nuclear translocation of P-Smad1/5/8 in the cytoplasm requires binding to Smad4. We found that, after Tmprss6 overexpression (Tmprss6 group), the expression of Smad4 in the cytoplasm was decreased, while the levels of the protein in the nucleus were increased compared with the Vector and WT groups (Fig. 2D, F). The decreased P-Smad1/5/8 levels in the nucleus inhibited pro-hepcidin expression (Fig. 2G, H). To investigate if the low levels of pro-hepcidin in neuro-2a cells regulated FPN1, possibly affecting intracellular iron content, we analyzed FPN1 levels by western blot analysis. As shown in Fig. 2G, H, FPN1 levels significantly increased in neuro-2a cells overexpressing Tmprss6 compared with the Vector and WT groups.

### Overexpression of Tmprss6 decreases intracellular iron content by increasing the expression of FPN1, thus inhibiting RNR activity and preventing progression to the cell cycle S phase

FPN1, as the only known iron-exporter protein, plays an important role in the regulation of intracellular iron [28]. As shown in Fig. 2I, J, compared to the control groups, the levels of FtH and FtL, the subunits of ferritin, a ubiquitous iron storage protein, were significantly decreased, while the levels of TfR1 protein, the gateway to cellular iron uptake, were significantly increased in neuro-2a cells with elevated Tmprss6. Consistent with these results, we found that the intracellular total iron content (Fig. 2K) and Fe^2+^ content (Fig. 2L, M) decreased significantly in the Tmprss6 group. Given these indication that the cells were iron starved, we proceeded to evaluate RNR activity to see if the low levels of iron limited RNR function. The RNR activity was indeed significantly decreased in the Tmprss6 group (Fig. 2N). The decrease of RNR activity coincided with cell cycle arrest in the Tmprss6 group, where there was a significant decrease in cells in the S phase compared with the Vector and WT groups (Fig. 2O, P). These results suggest that Tmprss6 can slow cell proliferation by decreasing their on available for RNR activity.

### Overexpression of Tmprss6 induces apoptosis in neuro-2a cells

**Fig. 3:**
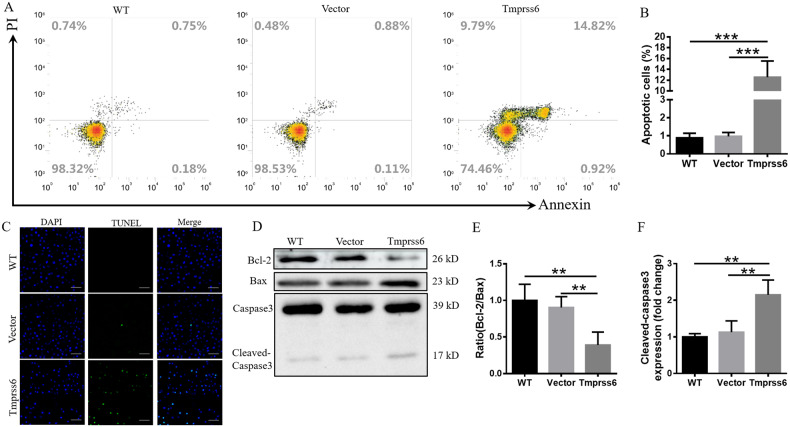
Overexpression of Tmprss6 induced apoptosis in neuro-2a cells. **A** Apoptosis, as measured by flow cytometry. **B** Proportion of apoptotic cells in the indicated groups. **C** Apoptosis induced by Tmprss6 overexpression by the TUNEL method, as described in the Methods section (Scale bar = 100 μm). **D** Levels of Bcl-2, Bax, and cleaved-caspase3, as assessed by western blot analysis (*n* = 4). **E**, **F** Quantification of Bcl-2/Bax **E** and Cleaved-caspase3 **F** expression from the experiment shown in **D**. Each sample’s relative expression level was calculated as described in the legend for Fig. 1D. Data are expressed as the mean ± SD. ∗∗*p* < 0.01, ∗∗∗*p* < 0.001.

In our cell culture experiments, we were surprised to find that Tmprss6 overexpression not only inhibited cell proliferation, but also stimulated cell death. Therefore, we proceeded to examine which forms of cell death, including apoptosis, necrosis, and ferroptosis, may have been occurring. Our results demonstrate that Tmprss6 overexpression was not associated with ferroptosis (Supplementary Fig. S2A, B) or necrosis (supplementary Fig. S2C, D), but was closely associated with apoptosis (Fig. 3). As shown in the annexin V assay results in Fig. 3A, B, the percentage of apoptotic cells was ~1% in the Vector and WT groups, while the Tmprss6 group was increased significantly, by about 12-fold. TUNEL assay also revealed that Tmprss6 overexpression caused a significant increase in apoptotic bodies compared to the Vector and WT groups (Fig. 3C). Finally, we evaluated the expression of Bcl-2, Bax, and cleaved-caspase3 to find that the ratio of Bcl-2/Bax ratio was significantly decreased (Fig. 3D, E), while cleaved-caspase3 levels (Fig. 3D, F) significantly increased in the Tmprss6 group, compared with the Vector and WT groups. Together these results indicate that the cell death caused by Tmprss6 overexpression was due to apoptosis.

### Tmprss6 overexpression-mediated apoptosis in neuro-2a cells is due to activation of the ATF3/P38 signaling pathway

**Fig. 4:**
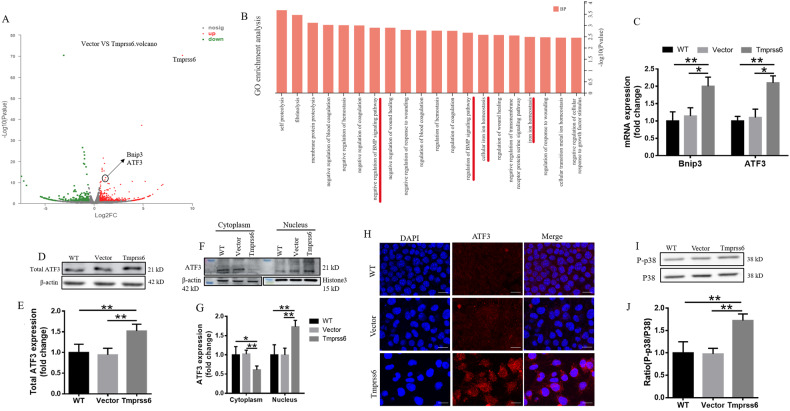
Activation of the ATF3/P38 signaling pathway in Tmprss6-overexpressing neuro-2a cells. **A** Volcano plot showing the significantly changed mRNAs in Tmprss6-overexpressing cells. **B** Pathway analysis was performed using GO data bases to identify functional enrichment based on the differentially expressed mRNA. **C** The ATF3 and Bnip3 expression was detected by qRT-PCR. **D** Levels of total ATF3, as assessed by western blot analysis (*n* = 4). **E** Quantification of total ATF3 expression from the experiment shown in **D**. **F** The levels of ATF3 in the cytoplasm and nucleus were detected by western blot analysis. **G** Quantification of ATF3 levels in the cytoplasm and nucleus from the experiment shown in **F**. **H** The subcellular localization of ATF3 in neuro-2a cells was determined by immunofluorescence (Scale bar = 20 μm). **I** The levels of P-p38 and p38 protein were detected by western blot analysis (*n* = 4). **J** Quantification of P-p38/p38 from the experiment shown in **I**. Each sample’s relative expression level was calculated as described in the legend of Fig. 1D. Data are expressed as the mean ± SD. ∗*p* < 0.05. ∗∗*p* < 0.01.

To explore how Tmprss6 overexpression induces apoptosis, we used RNA sequencing to screen for changes in apoptosis-stimulating gene expression. Compared with the Vector group, Tmprss6 overexpression significantly up-regulated the expression of ATF3 and Bnip3 in the volcano diagrams (Fig. 4A). The most closely associated with the changes in gene expression are Bmp signaling pathways in the GO analysis in Fig. 4B. We validated the changes in ATF3 and BCL2/ adenovirus E1B 19 kDa interacting 3 (Bnip3) gene expression by qRT-PCR (Fig. 4C). We also examined ATF3 protein levels and found them to be increased (Fig. 4D, E). To investigate whether overexpression of Tmprss6 causes nuclear translocation of ATF3, we performed western blot analysis on cytoplasmic and nuclear cell isolates. As shown in Fig. 4F, G, compared with the controls, there was a shift from the cytoplasm to the nucleus in the intracellular distribution of ATF3 after Tmprss6 overexpression. The increased nuclear translocation of ATF3 in the Tmprss6 group was also apparent in immunofluorescence experiments (Fig. 4H). We also found an increase in phosphorylated p38 (Fig. 4I, J)—the nuclear translocation of ATF3 is known to activate p38 mitogen-activated protein kinases, ultimately leading to apoptosis [29].

### Overexpression of Tmprss6 decreases iron levels to inhibit RNR activity and mediate apoptosis in SH-SY5Y cells

**Fig. 5:**
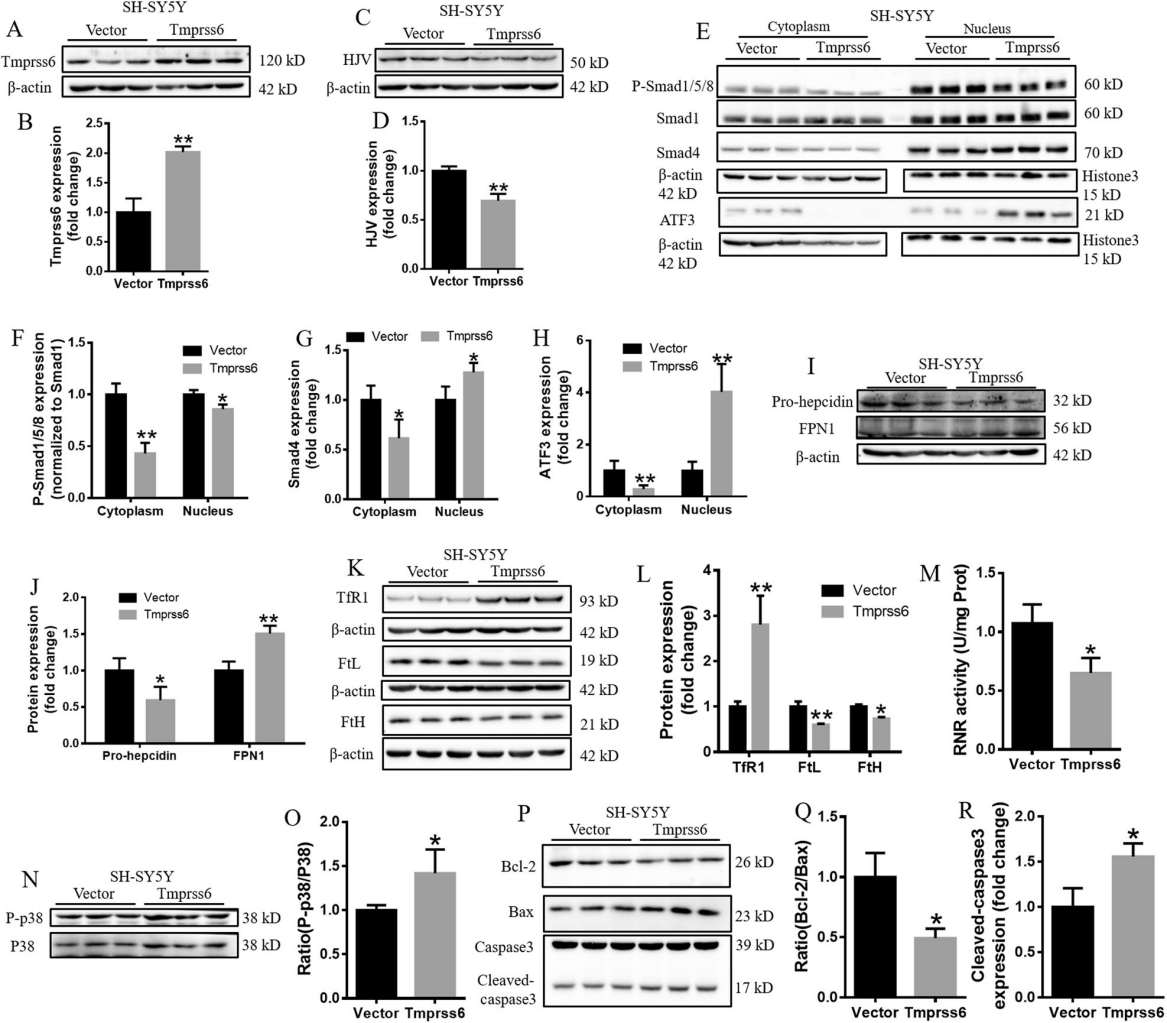
Overexpression of Tmprss6 decreases iron levels to inhibit RNR activity and mediate apoptosis in SH-SY5Y cells. **A** Western blot analysis of Tmprss6 expression in SH-SY5Y cells. **B** Quantification of Tmprss6 expression in SH-SY5Y cells. **C** Western blot analysis of HJV expression in SH-SY5Y cells. **D** Quantification of HJV expression in SH-SY5Y cells. **E** Expression of P-Smad1/5/8, Smad1, Smad4 and ATF3 proteins in the cytoplasm and nucleus, as detected by western blot analysis. Quantification of P-Smad1/5/8/Smad1 **F**, Smad4 **G**, and ATF3 **H** expression from the experiment shown in **E**. **I** Western blot analysis of pro-hepcidin and FPN1 levels in SH-SY5Y cells. **J** Quantification of pro-hepcidin and FPN1 expression from the experiment shown in I. **K** Western blot analysis of FtH, FtL, and TfR1 levels in SH-SY5Y cells. **L** Quantification of FtH, FtL and TfR1 levels from the experiment shown in **K**. **M** RNR activity, as tested using a kit from Mlbio. **N** Levels of P-p38 and p38 protein, as detected by western blot analysis. **O** Quantification of P-p38/p38 from the experiment shown in **N**. **P** Levels of Bcl-2, Bax and Cleaved-caspase3, as assessed by western blot analysis. **Q**, **R** Quantification of Bcl-2/Bax **Q** and Cleaved-caspase3 **R** expression from the experiment shown in **P**. The data are expressed as the mean ± SD, *n* = 3. ∗*p* < 0.05, ∗∗*p* < 0.01.

In addition, we also investigated the downstream effects of Tmprss6 activity in human neuroblastoma cell lines, SH-SY5Y. As shown in Fig. 5A, B, we overexpressed Tmprss6 (Tmprss6 group) in the SH-SY5Y cells. We then assessed the levels of HJV, P-Smad1/5/8, Smad4, ATF3, Pro-hepcidin, FPN1, TfR1, FtL, FtH, RNR activity, P-p38, Bcl-2, Bax and Cleaved-caspase3 (Fig. 5C–R) in the Tmprss6-overexpressing cells. Although the ratio of the changes was somewhat different, these results are consistent with our findings in neuro-2a cells overexpressing Tmprss6, demonstrating that the downstream effects of Tmprss6 activity in neuro-2a cells is also applicable to SH-SY5Y cells.

### Overexpression of Smad4 induces nuclear translocation of ATF3

**Fig. 6:**
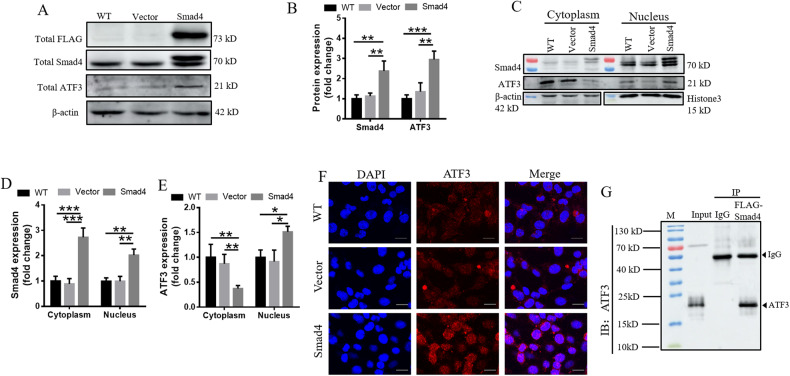
Nuclear translocation of ATF3 in Smad4-overexpressing neuro-2a cells. Neuro-2a cells were transfected with pcDNA3.1 (Vector group) or pcDNA3.1-Smad4-3Flag (Smad4 group) plasmids. **A** Levels of total FLAG, Smad4, and ATF3, as assessed by western blot analysis (*n* = 4). **B** Quantification of total Smad4 and ATF3 expression from the experiment shown in **A**. **C** The levels of Smad4 and ATF3 in the cytoplasm and nucleus were detected by western blot analysis (*n* = 4). **D**, **E** Quantification of Smad4 and ATF3 levels in the cytoplasm and nucleus in the experiment shown in E. Each sample’s relative expression level was calculated as described in the legend of Fig. 1D. **F** The subcellular localization of ATF3 in neuro-2a cells was examined by immunofluorescence (Scale bar = 20 μm). **G** The interaction between FLAG (Smad4) and ATF3 was examined by immunoprecipitation. Data are expressed as the mean ± SD. ∗*p* < 0.05.∗∗*p* < 0.01.***p < 0.001.

To explore the mechanism whereby Tmprss6 overexpression results in ATF3 nuclear translocation, we constructed a Smad4-FLAG overexpression plasmid to simulate the increased Smad4 expression in the nucleus by Tmprss6 overexpression (Tmprss6-FLAG is not expressed in the experiments in Fig. 6). As shown in Fig. 6A, B, compared with the controls, the increased expression of Smad4 was accompanied increased expression of ATF3. Overexpression of Smad4 also induced nuclear translocation of ATF3 compared to the Vector and WT groups (Fig. 6C–E). We confirmed this shift of ATF3 to the nucleus by immunofluorescence experiments (Fig. 6F). To explore the molecular mechanism of the ATF3 nuclear translocation caused by Smad4 overexpression, we performed immunoprecipitation experiments, the results of which suggest that an interaction between ATF3 and Smad4 occurs (Fig. 6G). Thus, ATF3 nuclear transposition may be the consequence of its binding to Smad4.

### Disruption of ATF3 expression alleviates apoptosis induced by overexpression of Tmprss6 in neuro-2a cells

**Fig. 7:**
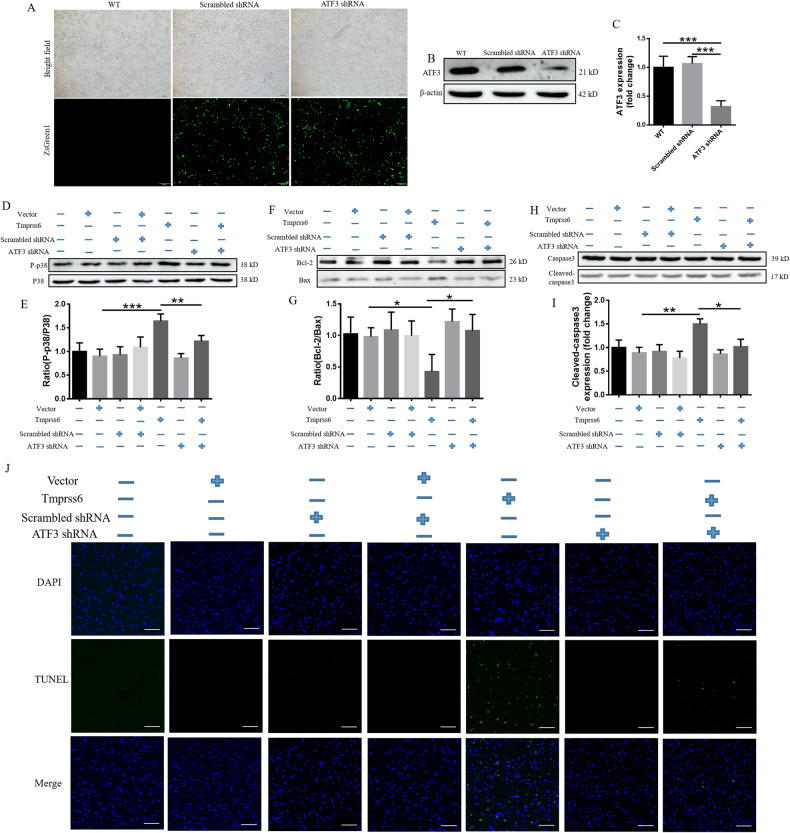
Effects of ATF3 expression on Tmprss6-induced apoptosis in neuro-2a cells. **A** Live ZSgreen1 imaging was performed 72 h after transfection with Scrambled shRNA or ATF3 shRNA plasmids. **B** Levels of ATF3, as assessed by western blot analysis (*n* = 4). **C** Quantification of ATF3 expression from the experiment shown in **B**. **D**–**I** Levels of P-p38 **D**, p38 **D**, Bcl-2 **F**, Bax **F**, and cleaved-caspase3 **H**, as assessed by western blot analysis (*n* = 4), from the first to the seventh lane are the WT group, Vector pcDNA3.1-transfected cells (Vector group), Scrambled shRNA-transfected cells (Scrambled shRNA group), pcDNA3.1 and Scrambled shRNA co-transfection group, Tmprss6-transfected cells (Tmprss6 group), ATF3 shRNA-transfected cells (ATF3 shRNA group), Tmprss6 and ATF3 shRNA co-transfection group. Quantification of P-p38/p38 **E**, Bcl-2/Bax **G**, and Cleaved-caspase3 **I** expression from the experiments shown in **D**, **F**, and **H**, respectively. Each sample’s relative expression level was calculated as described in the legend of Fig. 1D. **J** TUNEL method showing the induction of apoptosis in the indicated groups (Scale bar = 100 μm). Data are expressed as the mean ± SD. ∗*p* < 0.05. ∗∗*p* < 0.01, ∗∗∗*p* < 0.001.

To further confirm the role of ATF3 in Tmprss6 overexpression-mediated apoptosis, we inhibited the expression of ATF3 via shRNA. As shown in Fig. 7A–C, the expression of ATF3 was significantly inhibited in the ATF3-targeting short hairpin RNA (shRNA) group compared with the Scrambled shRNA and WT groups. Inhibition of ATF3 expression by the shRNA in Tmprss6-overexpressing cells decreased the ratio of P-p38/p38 (Fig. 7D, E) and the levels of cleaved-caspase3 (Fig. 7H, I), while the ratio of Bcl-2/Bax was elevated (Fig. 7F, G). TUNEL assay also revealed that inhibition of ATF3 expression in Tmprss6-overexpressing cells significantly reduced the formation of apoptotic bodies (Fig. 7J). These results confirm that ATF3 plays a central role in Tmprss6 overexpression-mediated apoptosis.

### Overexpression of Tmprss6 inhibits tumor growth and initiates apoptosis

**Fig. 8:**
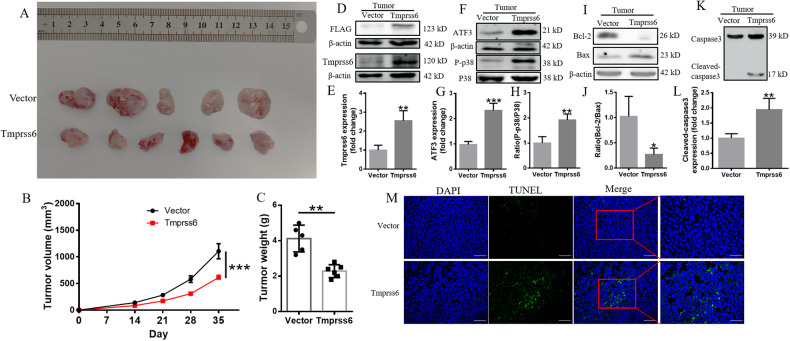
Effect of Tmprss6 overexpression on tumor growth and tumor cell apoptosis. **A** Photo of ex vivo tumors at 5 weeks post cell implantation in nude mice. **B** Tumor growth curves of nude mice within 5 weeks after cell implantation. **C** Tumor weights of nude mice at 5 weeks post cell implantation. **D**–**L** Levels of FLAG **D**, Tmprss6 **D**, ATF3 **F**, P-p38 **F**, p38 **F**, Bcl-2 **I**, Bax **I**, and Cleaved-caspase3 **K**, as assessed by western blot analysis (*n* = 4). Quantification of Tmprss6 **E**, ATF3 **G**, P-p38/p38 **H**, Bcl-2/Bax **J**, and Cleaved-caspase3 **L** expression from the experiments shown in **D**, **F**, **I**, and **K**, respectively. Each sample’s relative expression level was calculated as described in the legend of Fig. 1D. **M** TUNEL method showing the induction of apoptosis in the tumors in the indicated groups (Scale bar = 100 μm). Data are expressed as the mean ± SD. ∗*p* < 0.05. ∗∗*p* < 0.01, ∗∗∗*p* < 0.001.

To examine whether Tmprss6 overexpression can affect NB tumor growth, we subcutaneously implanted neuro-2a cells into nude mice. The tumors derived from cells overexpressing Tmprss6 grew at a significantly lower rate than those in the Vector group (Fig. 8A). We also measured the tumor growth, and the weights of the tumors. As shown in Fig. 8B, C, compared with the Vector group, all two of these values were significantly decreased in the Tmprss6 group, and the results of hematoxylin and eosin staining in tumor sections showed that the Vector group tumors are quite dense, while the tumor structure in the Tmprss6 group was looser and with lighter nuclear staining (Supplementary Fig. S3). We confirmed the continuous overexpression of Tmprss6 and FLAG in the process of tumor growth by western blot analysis (Fig. 8D, E). We also used western blot analysis to examine the levels of ATF3, P-p38, p38, Bcl-2, Bax, and cleaved-caspase3 in tumor tissue (Fig. 8F–L). Consistent with our cell culture experimental results, ATF3, P-p38, Bax, and cleaved-caspase3 expression increased significantly, while Bcl-2 expression decreased significantly in the Tmprss6 group. TUNEL staining further confirmed that overexpression of Tmprss6 induced the production of apoptotic bodies in tumor tissues (Fig. 8M). These results suggest that overexpression of Tmprss6 inhibits tumor growth and initiates apoptosis through the ATF3/P38 signaling pathway.

## Discussion

Proteolytic enzymes have long been thought to be involved in carcinogenesis, since proteolytic enzymes can hydrolyze the extracellular matrix (ECM), allowing cancer cells to escape the basement membrane and invade surrounding tissues [22]. Notably, cell surface proteases, such as Tmprss6, also activate a variety of growth factors and their associated receptors, which are essential for the activation of oncogenic signaling pathways [30–32]. So far, several members of the TTSP family have been linked to cancer progression [32–34].

Multiple clinical studies have shown that Tmprss6 levels are decreased in tumor progression, while low gene expression correlated with poor prognosis in triple-negative breast cancer [23, 35, 36]. Consistent with the clinical observations, overexpression of Tmprss6 has been found to inhibit the invasion and growth of breast and prostate cancer cells in both in vivo and in vitro experiments [36, 37]. Webb et al. [37] suggested that Tmprss6 may inhibit the development of prostate cancer cells by reducing the levels of β-catenin in the tumor cell membrane. Knockout of Tmprss6 at the cellular level resulted in increased levels of β-catenin, while overexpression of Tmprss6 had the opposite effect. Although Tmprss6 is well known for its association with some types of cancer, surprisingly little is known about the mechanisms by which it is involved in the development and growth of cancer, especially in the molecular control of the cell cycle and apoptotic processes in tumor tissues.

Here, we evaluated the role of Tmprss6 in a neuroblastoma cell line and its derived tumors. Since elevated Tmprss6 interfered with cell cycle progression and triggered apoptosis, we continued to test the overexpression model by investigating the mechanism of neuro-2a growth inhibition. Previous studies have shown that Tmprss6 cleaves HJV, a BMP co-receptor, on the surface of hepatocytes, modulating the BMP/SMAD signaling pathway that influences *HAMP* expression [26]. Consistent with this, we observed that Tmprss6 overexpression in neuro-2a cells reduced the level of HJV by cleaved HJV (Fig. 2A–C), thereby inhibiting the Bmp-Smad signaling pathway (Fig. 2D–F), and reducing the expression of pro-hepcidin (Fig. 2G, H). Meanwhile, we also tested the effects of Tmprss6 knockdown in neuro-2a cells, which significantly activated the Bmp-Smad signaling pathway (Supplementary Fig. S4). The decreased expression of pro-hepcidin decreased the total intracellular iron and Fe^2+^ content by increasing the level of FPN1 (Fig. 2G–M). The decrease in Fe^2+^ content inhibited the activity of RNR, thus arresting the cell cycle ahead of the S phase, ultimately leading to inhibited tumoral cell proliferation (Fig. 2N–P).

Apoptosis plays a key role in the pathogenesis of numerous diseases [38]. In neurodegenerative diseases, pathogenesis entails an excess of apoptosis [39], whereas in cancer, too little apoptosis can be the culprit, enabling the expansion of neoplastic cells [40]. The mechanism of apoptosis is complex and involves several pathways. Importantly, apoptosis is an important target in the treatment of cancer [41]. In our study, we found that Tmprss6 overexpression in neuro-2a cells leads to a decrease in proliferation by stimulating apoptosis (Fig. 3), without affecting ferroptosis or necrosis (supplementary Fig. S2). RNA sequencing revealed that Tmprss6 overexpression led to increased levels of ATF3 and Bnip3, while GO analysis showed functional enrichment of Bmp pathways (Fig. 4A–B). Meanwhile, we confirmed there was increased expression of ATF3 and Bnip3 (Fig. 4C–E). Elevated ATF3 levels promote nuclear translocation and activate downstream signaling via phosphorylation of p38, which mediates apoptosis (Fig. 4F–J).

We also found that the expression and nuclear translocation of ATF3 was increased in neuro-2a cells when Smad4 was overexpressed (Fig. 6A–F), which is likely the result of an interaction between the two proteins, as we demonstrated by immunoprecipitation experiments (Fig. 6G). Thus, Smad4 not only assists in the nuclear translocation of P-Smad1/5/8, but also stimulates the nuclear translocation of ATF3. We conjecture that since the overexpression of Tmprss6 inhibits the phosphorylation of Smad1/5/8, the amount of Smad4 bound to P-Smad1/5/8 is accordingly decreased, freeing up Smad4 to promote an increased nuclear translocation of ATF3.

**Fig. 9:**
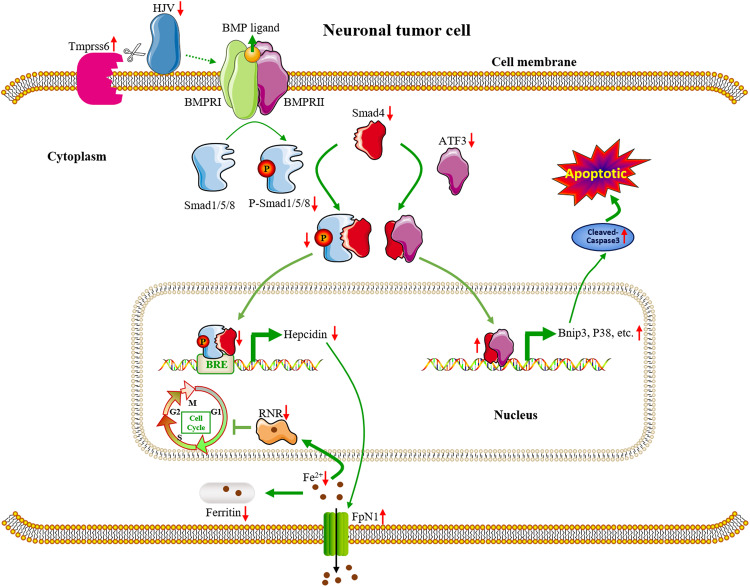
Schematic diagram of a working model of the inhibition of tumor growth by Tmprss6 overexpression. The overexpression of Tmprss6 reduces HJV levels by cleaving HJV, thereby inhibiting the phosphorylation of Smad1/5/8 and retention of the latter in the cytoplasm. Due to the decreased P-Smad1/5/8 levels, the transport of P-Smad1/5/8 to the nucleus by Smad4 is reduced. Decreased P-Smad1/5/8 in the nucleus inhibits pro-hepcidin expression. The decreased pro-hepcidin levels block the internalization and degradation of FPN1. The increased FPN1 level leads to the export of cellular Fe^2+^, limiting the activity of RNR, ultimately arresting the cell cycle before the S phase. Additionally, the decreased amount of P-Smad1/5/8 frees up Smad4 to bind to ATF3 and translocate it to the nucleus, thus activating p38 signaling and the expression of Bnip3, leading to apoptosis.

Figure 9 presents a schematic representation of the possible mechanism of Tmprss6-mediated inhibition of tumor growth. We propose Tmprss6 as a new candidate target for inhibiting neuronal tumor cell proliferation and mediating apoptosis in cancer.

## Materials and methods

### Reagents

The following reagents were used: Minimum Essential Medium (Invitrogen, Carlsbad, CA, USA), fetal calf serum (Invitrogen, USA), Nonessential Amino Acid Solution (Invitrogen, USA), TRIzol reagent (15596018, Invitrogen, USA), Tris and Glycine (Amresco, Washington, USA), Reverse Transcriptase MMLV, dNTP Mixture and Recombinant RNase Inhibitor (TaKaRa, saka-shi, Japan). The following antibodies were used: β-actin (1:10000, cw0096m, CWbio, Beijing, China), FtL (1:5000, ab109373, Abcam, SF, CA, USA), FtH (1:5000, ab183781, Abcam, USA), Hepcidin (1:5000, ab30760, Abcam, USA), Tmprss6 (1:10000, 12950-1-AP, Proteintech, Wuhan, China), FLAG (1:20000, 80010-1-RR, Proteintech, China), HJV (1:5000, 11758-1-AP, Proteintech, China), P-Smad1/5/8 (1:1000, #9516, Cell Signaling Technology, St. Louis, MA, USA), Smad1 (1:1000, #6944, Cell Signaling Technology, USA), Smad4 (1:1000, #46535, Cell Signaling Technology, USA), FPN1 (1:5000, MTPP11-S, ADI, San Antonio, Texas, USA), TfR1(1:5000, 13-6800, Invitrogen, USA), Bcl-2 (1:5000, 26593-1-AP, Proteintech, China), Bax (1:5000, 50599-2-Ig, Proteintech, China), Caspase3 (1:3000, #9662 S, Cell Signaling Technology, USA), Cleaved-caspase3 (1:3000, #9664, Cell Signaling Technology, USA), ATF3 (1:1000, ABP55330, Abbkine, Wuhan, China), P-p38 (1:2000, #4511, Cell Signaling Technology, USA), P38 (1:2000, #8690 S, Cell Signaling Technology, USA), ACSL4 (1:5000, ab155282, Abcam, USA), GPX4 (1:5000, ab125066, Abcam, USA), RIP1 (1:5000, 17519-1-AP, Proteintech, China), RIP3 (1:5000, 17563-1-AP, Proteintech, China), protein marker (26617, Thermo, Carlsbad, CA, USA), anti-rabbit IgG (1:20000, RPN4301, Amersham, London, UK), anti-mouse IgG (1:20000, RPN4201, Amersham, UK).

### Cell culture

Neuro-2a (ATCC, NO. CCL131, WT group), SH-SY5Y (ATCC, NO. CRL2266), Vector pcDNA3.1-transfected cells (Vector group), Tmprss6-transfected cells (Tmprss6 group), Smad4-transfected cells (Smad4 group), Scrambled shRNA-transfected cells (Scrambled shRNA group), ATF3 shRNA-transfected cells (ATF3 shRNA group) and Tmprss6 shRNA-transfected cells (Tmprss6 shRNA group) were maintained in MEM supplemented with fetal calf serum (10%, vol/vol), nonessential amino acids (0.1 mM), glucose (4.5 mg/ml), penicillin (100 U/ml), and streptomycin (100 mg/ml) in humidified 5% CO_2_ and 95% air at 37 °C. Vector and Tmprss6 cells were maintained in G418 (500 μg/ml) to select stable Tmprss6-transfected neuro-2a cells.

### Cell transfection

Efficient cell transfection experiments were performed using Lipofectamine^TM^ 3000 kits (L3000015, Invitrogen, USA), according to the manufacturer’s instructions. Briefly, neuro-2a cells were first inoculated in six-well plates and allowed to grow to a density of 70%–90%. Next, the plasmid DNA–liposome complexes were prepared, 2 μl Lipofectamine^TM^ 3000 and 4 μl P3000 were added to every 2 μg of plasmid DNA, and then diluted with Opti-MEM medium. Finally, the DNA–liposome complex was added to the neuro-2a cells, which were placed in a 37 °C, 5% CO_2_ tissue culture incubator for generally 48–72 h before evaluation for transfected gene expression.

### Immunofluorescence assay

Cells were fixed in 4% paraformaldehyde for 1.5–2 h, the fix solution was discarded, and the cells were washed 3 times with 0.01 M phosphate-buffered saline (PBS) for 5 min. A 0.5% Triton-100 solution was then applied for a 10 min treatment, after which the samples were washed twice with 0.01 M PBS. Goat serum (diluted 1:10 with PBS) was added, after which the samples were incubated at 37 °C for 50 min. The primary antibody, diluted in PBS, was added drop-wise, and the samples were incubated at 4 °C overnight. The samples were returned to room temperature for 15 min and washed 3 times with 0.01 M PBS for 5 min. Rhodamine-labeled, goat anti-rabbit secondary antibodies (1:200) were added, and the samples were incubated at room temperature for 90 min. The samples were then washed 4 times with 0.01 M PBS for 5 min. DAPI (1:1000, diluted with PBS; 4 min) was used to stain the nuclei. Excess DAPI was removed by washing 6 times with 0.01 M PBS for 5 min. Images were acquired using a fluorescence confocal microscope (Olympus FV3000, Japan).

### Western blot

For the extraction of total protein from tumor tissues from nude mice or neuro-2a cells, the samples were first placed into RIPA buffer and centrifuged at 12,000 × *g* for 20 min. For the nuclear and cytoplasmic protein isolation from neuro-2a cells, a nuclear and cytoplasmic protein extraction kit (P0027, Beyotime, Shanghai, China) was used according to the manufacturer’s instructions. Briefly, the cell samples were added to cytoplasmic protein extraction reagent A, violently shaken for 5 s, placed in an ice bath for 10–15 min, added to cytoplasmic protein extraction reagent B, violently shaken for 5 s, and placed in an ice bath for 1 min. After centrifugation at 12,000 × *g* for 5 min, the supernatant contained the cytoplasmic proteins. Nuclear protein extraction reagent was added to the precipitate, which was violently shaken for 15–30 s, placed in an ice bath for 2 min, and centrifuged at 12,000 × *g* for 10 min; the supernatant contained the nuclear protein. The protein supernatant in the above process was collected and quantified using a BCA kit (Kang Wei, China). The samples were resolved by SDS-PAGE (10–12% acrylamide), and then transferred to nitrocellulose membranes (Millipore, Bedford, MA, USA). The membranes were blocked with 5% skim milk in TBS-T for 1.5 h and then incubated with primary antibodies overnight at 4 °C. The membranes were washed with TBS-T buffer and then incubated for 90 min at room temperature with anti-rabbit or anti-mouse IgG conjugated with horseradish peroxide. After washing, immune reactive proteins were detected by the enhanced chemiluminescence (ECL) method.

### Quantitative real-time reverse transcription-PCR (qRT-PCR)

Neuro-2a cells were homogenized with TRIzol reagent, extracted with chloroform, and precipitated with isopropyl alcohol, according to the manufacturer’s instructions. RNA was reverse transcribed with MMLV reverse transcriptase and Oligo-dT primers after being washed twice with 75% alcohol. SYBR green PCR Master Mix was used for PCR amplification. The cycle threshold (Ct) value for a given gene of interest was first normalized to β-actin in the same sample, and then the relative differences between the control and each of the other groups were calculated using equation 2^−ΔΔCt^, and expressed as relative fold changes of the control group. The primer sequences used for amplification were as follows:

Tmprss6 forward: 5’- TTGCTGGTCTTGGCTGCGCT-3’

Tmprss6 reverse: 5’-AATGACGGTTGAGCACCCGGAG-3’

ATF3 forward: 5’-GCCAAGTGTCGAAACAAGAAAAAG-3’

ATF3 reverse: 5’-TCCTCGATCTGGGCCTTCAG-3’

Bnip3 forward: 5’-CCTGTCGCAGTTGGGTTC-3’

Bnip3 reverse: 5’-GAAGTGCAGTTCTACCCAGGAG-3’

β-actin forward: 5’- AGGCCCAGAGCAAGAGAGGTA -3’

β-actin reverse: 5’-TCTCCATGTCGTCCCAGTTG -3’

### Immunoprecipitation (IP)

Non-denatured lysate (P0013, Bytotime, China) was added to the cell samples, which were then placed in an ice bath for 10 min and centrifuged (12,000 × *g*). The supernatants were collected, protein A/G beads (coupled with FLAG or an IgG antibody) were added to the supernatants, and the samples were slowly shaken at 4 °C in a silent mixer overnight. On the second day after the immunoprecipitation reaction, the protein A/G beads were centrifuged for 5 min at 12,000 × *g*, and washed 3 times with pre-cooled PBS. After adding SDS-PAGE loading buffer, the samples were incubated in a 95 °C water bath 5 min and centrifuged. Finally, the supernatants were collected for western blot analysis.

### Measurement of total cellular iron levels by ICP-MS

Total cellular iron levels were measured by ICP-MS using a previously described method [42]. Briefly, the cell samples were thermally digested in 70% nitric acid using a microwave method at an asymptotic temperature. After the digested samples were diluted, an Agilent 7500ce ICP-MS (Agilent Technologies, Santa Clara, CA) was used to determine the total iron content of the samples. An 8-point calibration curve was performed before sample analysis. At least 3 samples of each cell preparation were analyzed by ICP-MS. The total iron content of the sample was calculated by dry sample weight.

### Fe^2+^ content

Cytoplasmic ferrous iron content was assessed using FerroOrange (DojinDo, Kyushu Island, Japan). The assay does not detect ferric iron that is bound to proteins. After reduction to the ferrous form (Fe^2+^), cytoplasm Fe^2+^ (Cyto-Fe) reacts with probes to produce a stable colored complex. The cells were counterstained with Hoechst (1:1000, diluted with PBS) for 30 min at 37 °C. After washing the samples 3 times for 5 min with 0.01 M PBS, the fluorescence intensity was analyzed using a confocal microscope (Olympus FV3000, Japan).

### RNR activity assay

RNR activity in neuro-2a cells was carried out using a kit from Mlbio company (NO. YJ151420, Shanghai, China), according to the manufacturer’s instructions.

### Assessment of apoptosis by flow cytometry and TUNEL staining

TUNEL detection was performed using a TUNEL FITC Apoptosis Detection Kit (Vazyme Biotech CO., Nanjing, China), according to the manufacturer’s instructions. Briefly, tissue slides or neuro-2a cells were pretreated with 10 μg/ml proteinase K for 10 min and then incubated with the reaction mixture containing terminal deoxynucleotidyl transferase (TdT) and fluorescein-conjugated deoxyuridine triphosphate (dUTP) for 1 h at 37 °C. The nuclei were counterstained with DAPI, and images were acquired using a confocal microscope (Olympus FV3000, Japan).

Apoptosis was detected using a FITC-Annexin V apoptosis assay kit (#C1062L, Beyotime, China), according to the manufacturer’s instructions. Neuro-2a cells were collected and stained with annexin V at 37 °C for 10 min. Next, the samples were centrifuged at room temperature at 1000 × *g* for 5 min. After washing the cells twice with PBS, the samples were stained with propidium iodide (PI). The percentage of apoptotic cells was analyzed by flow cytometry (CytoFLEX, Beckman Coulter).

### Cell cycle analysis

Cell cycle was assessed using a Cell Cycle Analysis Kit (#C1052, Beyotime, China), according to the manufacturer’s instructions. Neuro-2a cells were collected and stained with PI at 37 °C for 30 min, after which the samples were centrifuged at room temperature at 1000 × *g* for 5 min. The cells were washed twice with PBS, and the percentage of cells in each cell cycle was analyzed by flow cytometry (CytoFLEX, Beckman Coulter).

### RNA sequencing

Total RNA was extracted using TRIzol. The mRNA was sequenced on the Illumina Hiseq platform. Differential expression analysis of experimental and control groups was performed using the DESeq2 R package (1.16.1). The data were transformed into a volcano plot. Gene Ontology (GO) analysis of differentially expressed genes was implemented using the cluster Profiler R package.

### Allografts tumor growth in nude mice

Male, athymic Balb/c nu/nu mice, 4 weeks of age and free of specific pathogens, were acquired from Vital River Laboratory Animal Technology (Beijing, China). The mice were placed in sterile, microisolated cages in a 12-hour light/dark cycle environment in a specific pathogen-free facility. The animals had free access to pathogen-free water and food. 1 × 10^7^ tumoral cells/ml (in 0.2 ml PBS) were injected subcutaneously into the mice. After becoming visible, tumor growth was observed weekly. Five weeks after injection, the mice were humanely killed, and the primary tumor volumes and weights were measured.

### Statistical analysis

All experiments were performed at least in triplicate. Statistical analyses were conducted using GraphPad Software’s Prism 7 (GraphPad Software, USA). The values are reported as the mean ± SD. Two-group comparisons were conducted using the Student’s *t* test (two-tailed), while multi-group comparisons were conducted by One-way ANOVA with Tukey’s post hoc analysis. *P* values < 0.05 were considered statistically significant.

### Supplementary information

SUPPLEMENTAL MATERIAL

Original Data File

The reproducibility checklist

## Data Availability

Additional data can be found in the Supplementary materials. The remaining datasets and material generated in the study are available from the corresponding authors upon reasonable request.
