# Supplementary material for: Transmembrane serine protease 6, a novel target for inhibition of neuronal tumor growth
Source: Cell Death Dis. 2024 Jan 13;15(1):49. doi: 10.1038/s41419-024-06442-x (PMC10787746; doi:10.1038/s41419-024-06442-x)
Supplement: Supplementary file 1 — SUPPLEMENTAL MATERIAL [file 41419_2024_6442_MOESM1_ESM.docx]

**Supplementary Materials and Methods:**

**Plasmid constructs**

The mouse Tmprss6 cDNA coding sequence (CDS) was amplified by polymerase chain reaction (PCR) using 5’-ATGCCGAGATGTTTCCAGCT -3’ and 5’- GGTCAGCACCTGCTGGATCCA-3’ primers and cloned into the pcDNA3.1 (+) vector. The 7930bp plasmid (Fig. S1A) contained a CMV promoter, the entire mouse Tmprss6 sequence (NCBI Reference Sequence: NM_027902.2), a C-terminal 3FLAG tag and the G418 screening marker. The forward primer, 5’-ATGGACAATATGTCTATAAC-3’, and reverse primer, 5’-AGGTTCCACCCACGGACCC-3’, were used to clone the entire mouse Smad4 (NCBI Reference Sequence: NM_001364967.1) coding sequence and connect it to the pcDNA3.1(+) plasmid. The total length of the plasmid was 7101bp (Fig. S1B). The construct sequences were confirmed by sequencing (GENEWIZ, Suzhou, China).

We generated a pLVX vector carrying an ATF3 shRNA sequence, custom-designed using online software available from Invitrogen and TaKaRa. The ATF3 shRNA sequence was: 5’-GGAGATGTCAGTCACCAAGTCTTCAAGAGAGACTTGGTGACTGACATCTCCTTTTTT-3’.A nonsense shRNA sequence (5’-GTCGAACACCAATATACGA-3’) was also prepared and inserted into the pLVX vector for use as a negative control (Scrambled shRNA). The plasmid was 9044bp in length (Fig. S1C), and included a U6 promoter, the ATF3 shRNA sequence, and ZSgreen1 fluorescent protein. Constructs sequences were confirmed by sequencing (GENEWIZ, Suzhou, China). Meanwhile, we also generated a pLVX vector carrying Tmprss6 shRNA sequence, The Tmprss6 shRNA sequence was: 5’-GGTATTTCCTAGGGTACAAAGTTCAAGAGACTTTGTACCCTAGGAAATACCTTTTTT-3’.

**H & E staining**

All staining was performed at room temperature (24 ° C). Briefly, tumor sections were stained in Harris’ hematoxylin for 6 minutes, samples were washed in distilled water for 5 minutes, then differentiated in acidic ethanol for 1-3 seconds, samples were washed in distilled water for 5 minutes, stained with 1% (w/v) eosin for 2 minutes, Then the samples were washed in running tap water and dehydrated through the alcohols and cleared in xylene Finally, wrap the slices in Canadian balsam.

**Supplementary Figures and Figure Legends:**


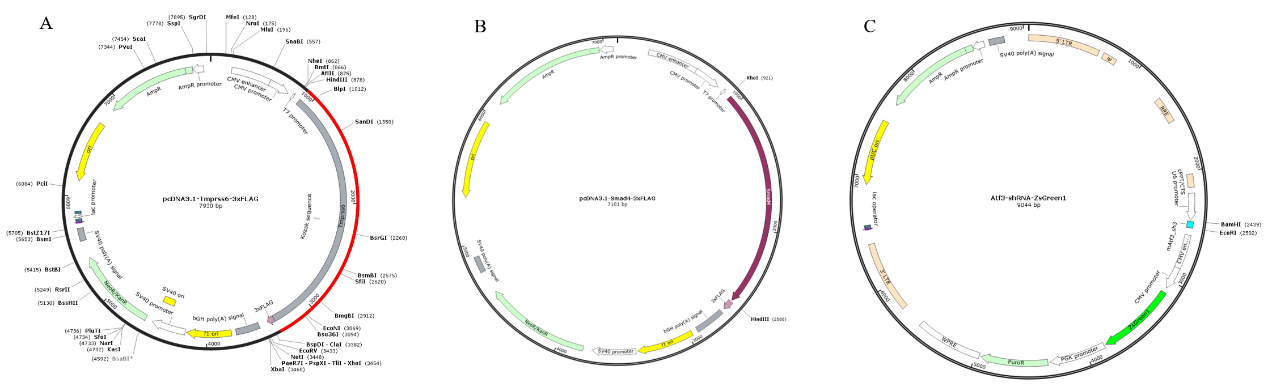


**Figure S1. Map of the plasmid**

(A) Map of the Tmprss6 overexpression plasmid (pcDNA3.1-Tmprss6-3FLAG). (B) Map of the Smad4 overexpression plasmid (pcDNA3.1-Smad4-3FLAG). (C) Map of the ATF3 shRNA plasmid (ATF3-shRNA-ZSgreen1).


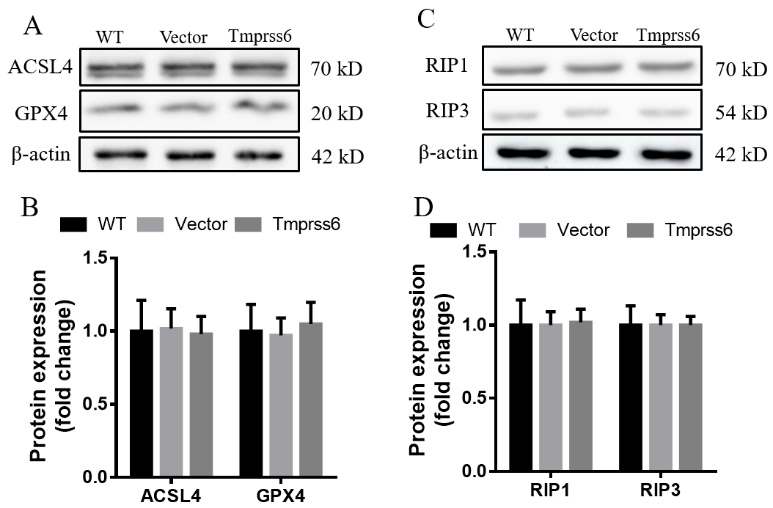


**Figure S2. Tmprss6 overexpression did not affect ferroptosis and necrosis of neuro-2a cells**

(A) The expression levels of ferroptosis marker ACSL4 and GPX4 were detected by Western blot. (B) Quantification of ACSL4 and GPX4 expression from the experiment shown in panel A. (C) The expression levels of necrosis marker RIP1 and RIP3 were detected by Western blot. (D) Quantification of RIP1 and RIP3 expression from the experiment shown in panel C. Data were expressed as mean ± SD, n =3.


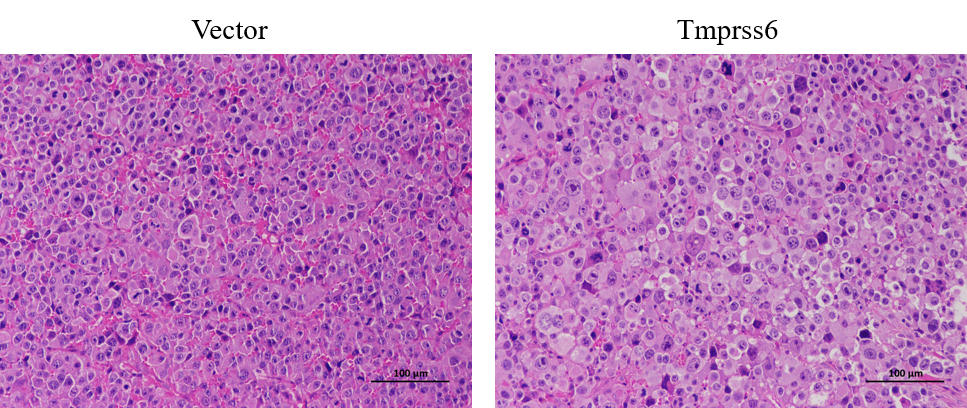


**Figure S3. Effect of Tmprss6 overexpression on tumor cell morphology**


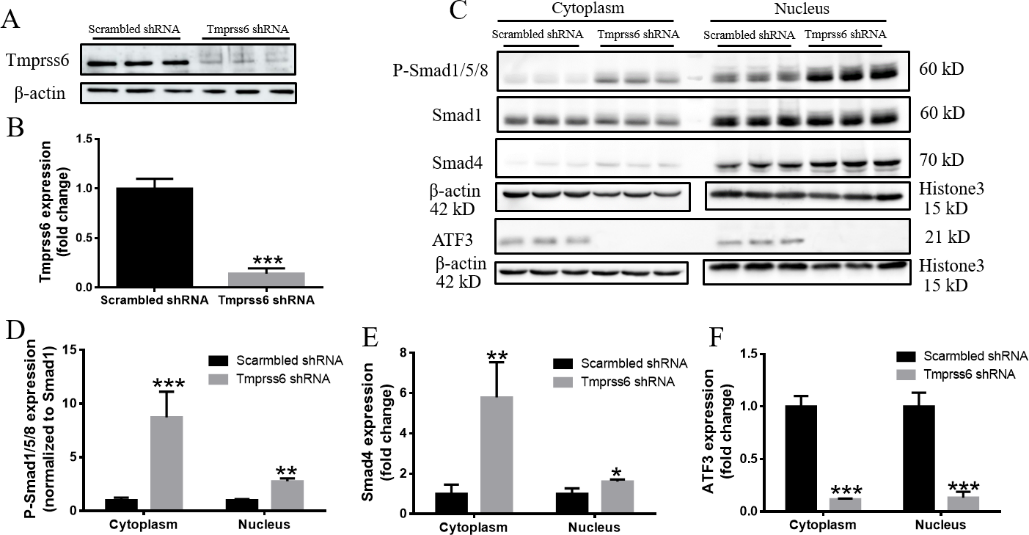


**Figure S4. Knocking down Tmprss6 in neuro-2a cells can significantly activate the Bmp-Smad signaling pathway**

(A) The expression levels of Tmprss6 was detected by Western blot. (B) Quantification of Tmprss6 expression from the experiment shown in panel A. (C) The expression of P-Smad1/5/8, Smad1, Smad4 and ATF3 proteins in the cytoplasm and nucleus were detected by western blot analysis. Quantification of P-Smad1/5/8 / Smad1 (D), Smad4 (E) and ATF3 (F) expression from the experiment shown in panel C. Data were expressed as mean ± SD, n =3. ∗p < 0.05. ∗∗p < 0.01, ∗∗∗p < 0.001.
