# Supplementary material for: Transmembrane serine protease 6, a novel target for inhibition of neuronal tumor growth
Source: Cell Death Dis. 2024 Jan 13;15(1):49. doi: 10.1038/s41419-024-06442-x (PMC10787746; doi:10.1038/s41419-024-06442-x)

Supplementary materials: full image scans of western blot membranes

Figure 1 B FLAG

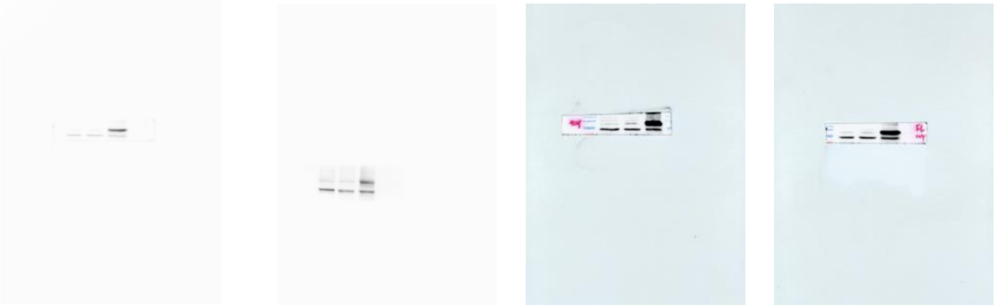

Figure 1 B β-actin to FLAG

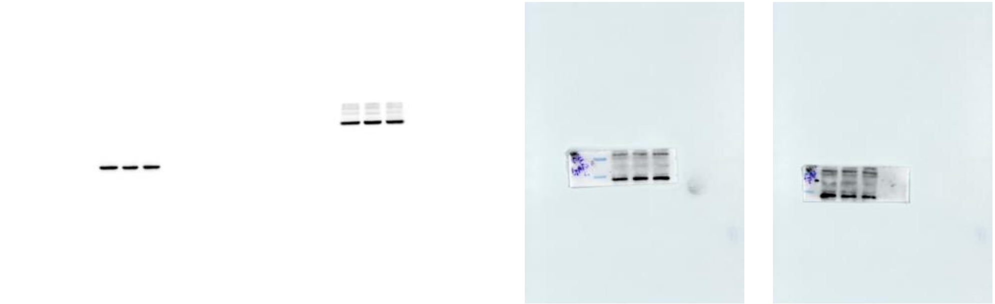

Figure 1 C Tmprss6

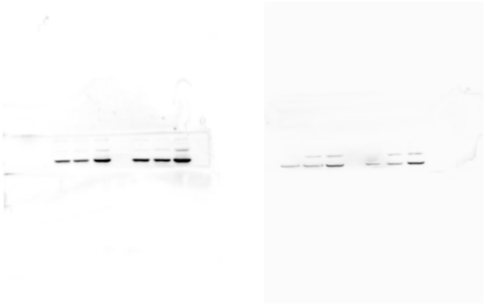

Figure 1 C β-actin to Tmprss6

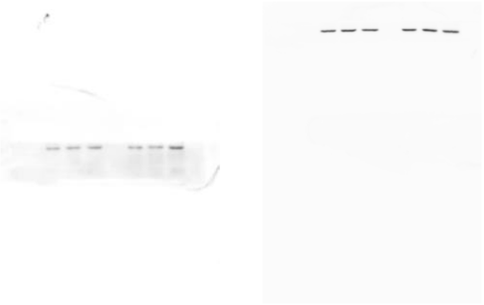

Figure 2 B HJV

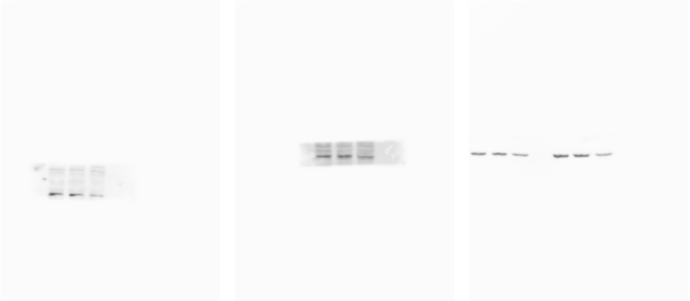

Figure 2 B β-actin to HJV

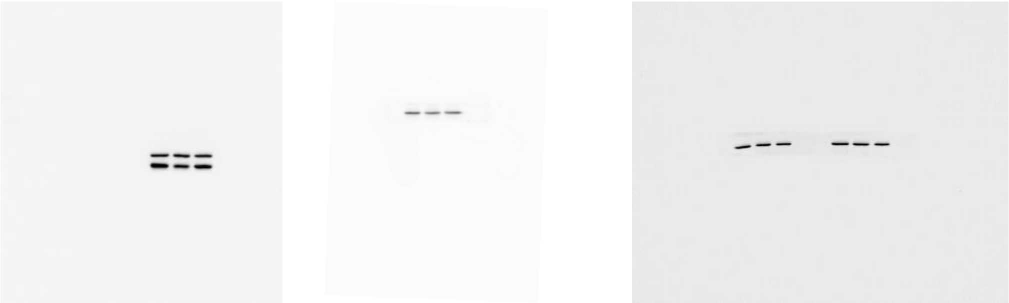

Figure 2 D P-Smad1/5/8

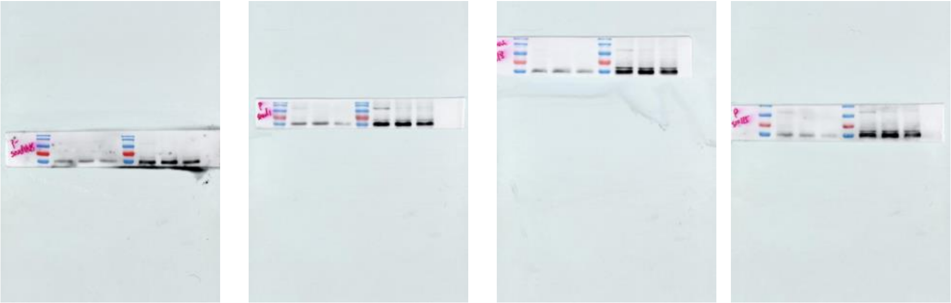

Figure 2 D Smad1 to P-Smad1/5/8

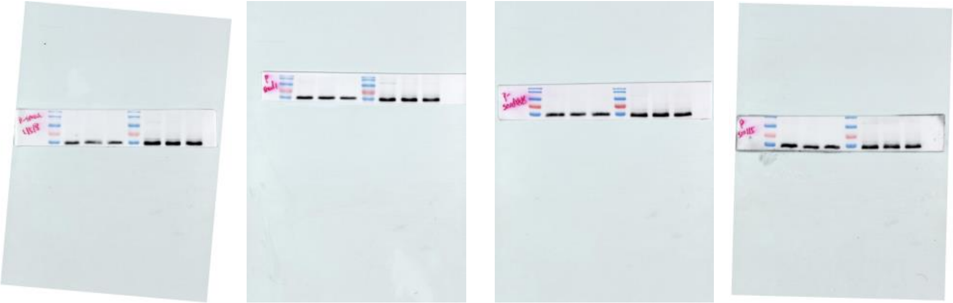

Figure 2 D Smad 4

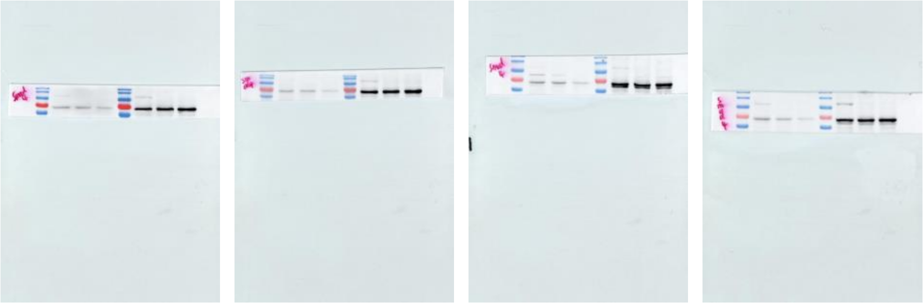

Figure 4 F ATF3

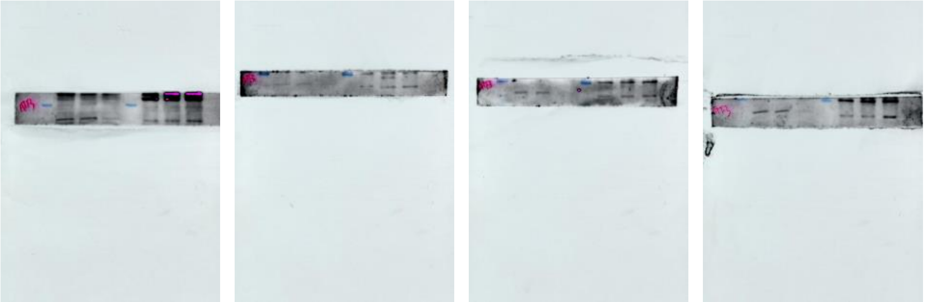

Figure 2 D β-actin to Figure 2 D Smad4 and Figure 4 F ATF3

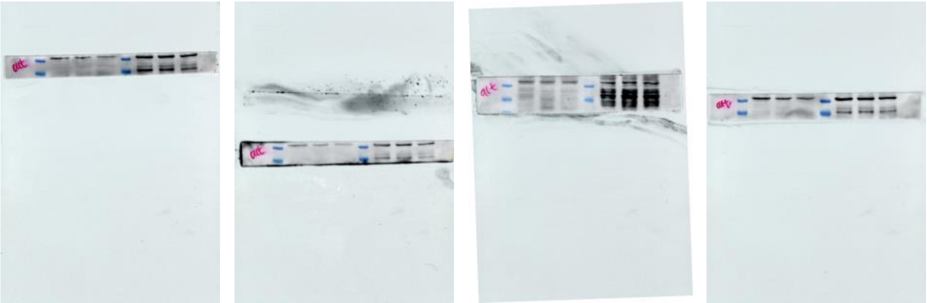

Figure 2 D Histone3 to Figure 2 D Smad4 and Figure 4 F ATF3

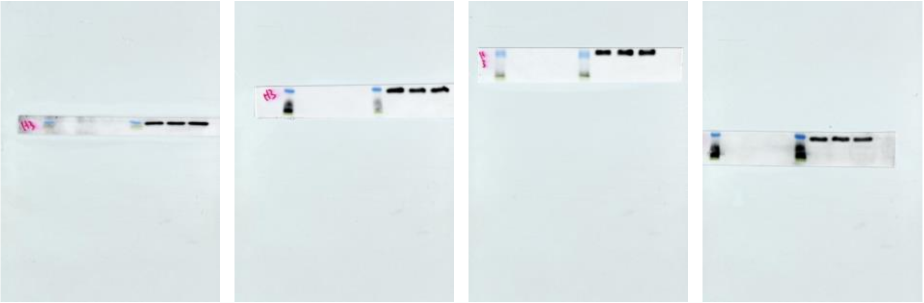

Figure 2 G Pro-hepcidin

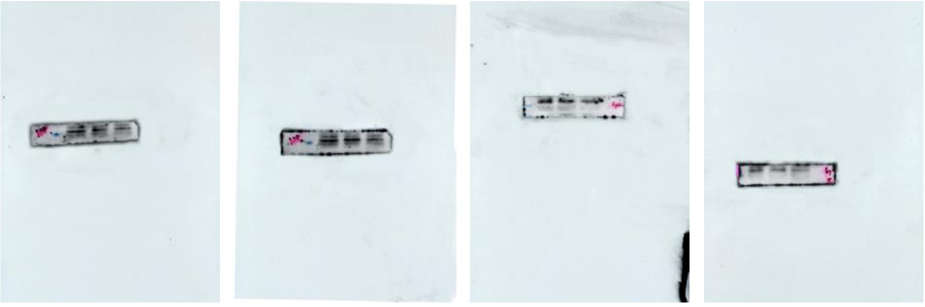

Figure 2 G β-actin to Pro-hepcidin

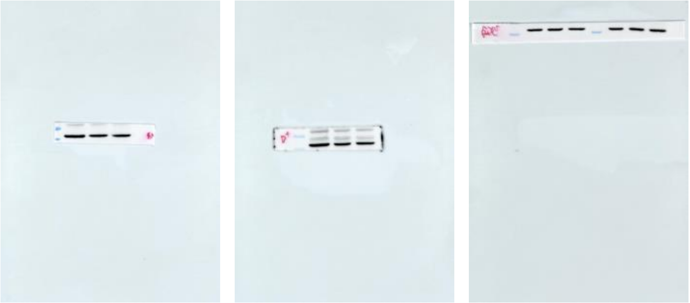

Figure 2 G FPN1

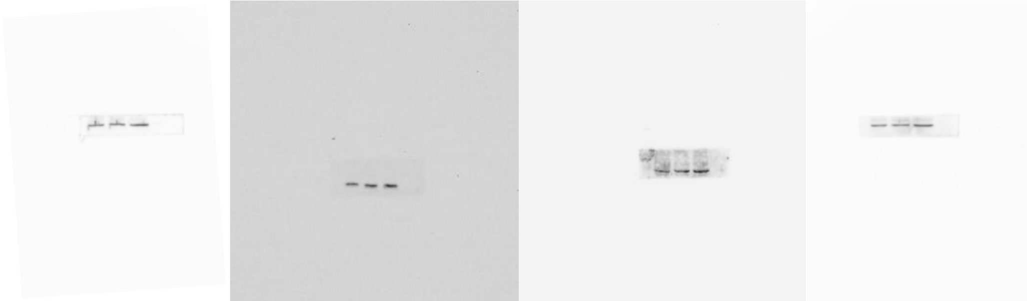

Figure 2 G β-actin to FPN1

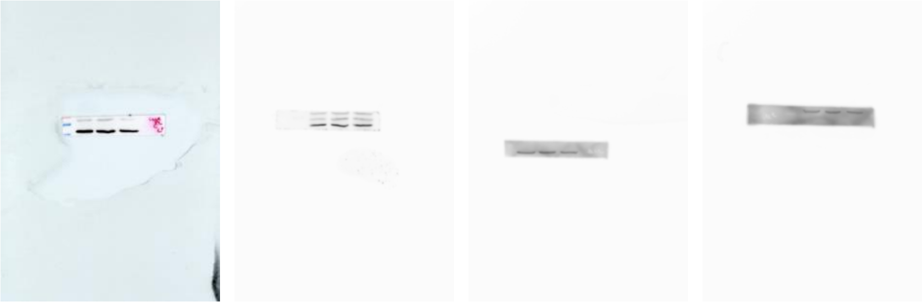

Figure 2 I TfR1

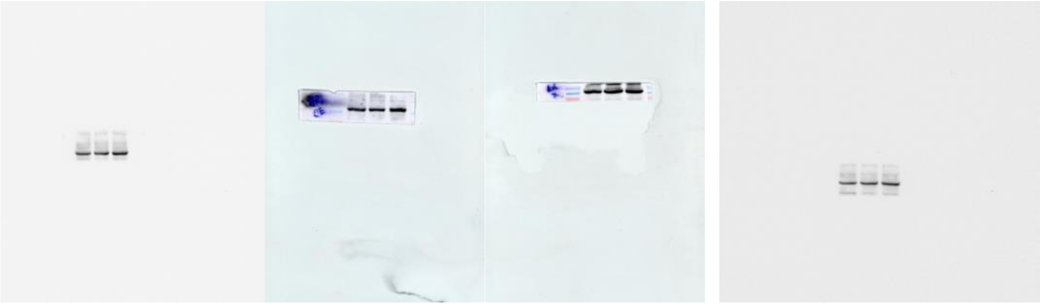

Figure 2 I FtL

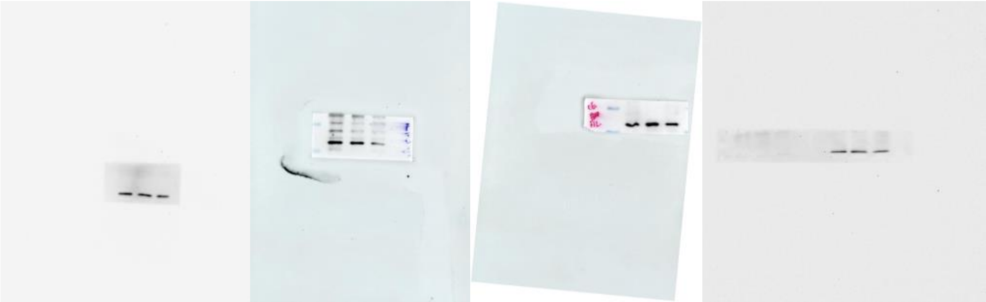

Figure 2 I FtH

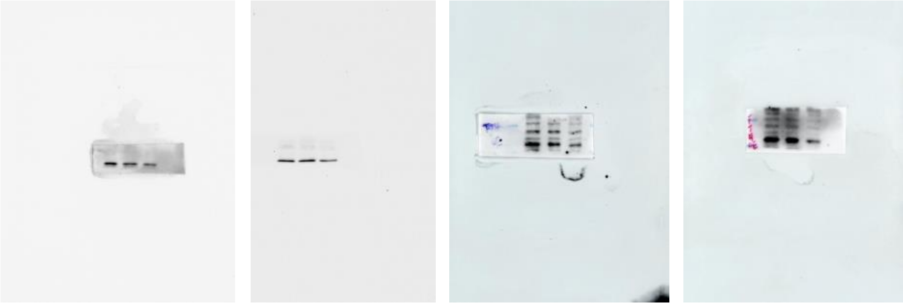

Figure 2 I β-actin to TfR1, FtL and FtH

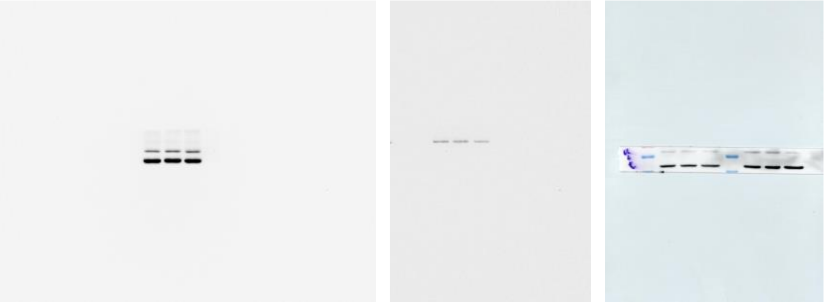

Figure 3 D Bcl-2（**Membrane 1**, From left to right: WT, Vector, Tmprss6. **Membrane 2**, From left to right: WT 2, WT 3, WT 4, Vector 2, Vector 3, Vector 4, Tmprss6 2, Tmprss6 3, Tmprss6 4.）

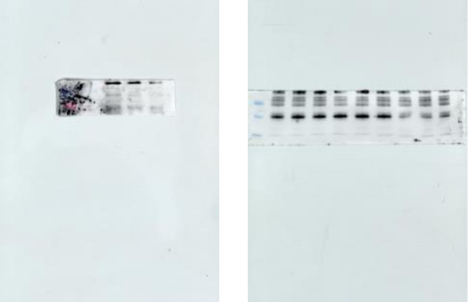

Membrane 1 **Membrane 2**

Figure 3 D Bax （**Membrane 1**, From left to right: WT, Vector, Tmprss6. **Membrane 2**, From left to right: WT 2, WT 3, WT 4, Vector 2, Vector 3, Vector 4, Tmprss6 2, Tmprss6 3, Tmprss6 4.）

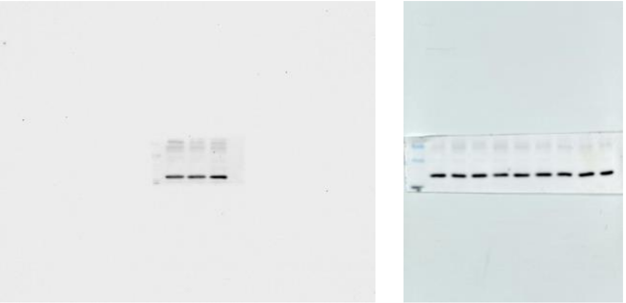

Membrane 1 **Membrane 2**

Figure 3 D Caspase3 and Cleaved-Caspase3（**Membrane 1**, From left to right: WT, Vector, Tmprss6. **Membrane 2**, From left to right: WT 2, WT 3, WT 4, Vector 2, Vector 3, Vector 4, Tmprss6 2, Tmprss6 3, Tmprss6 4. Membrane 3 is the image of membrane 2 after overexposure）

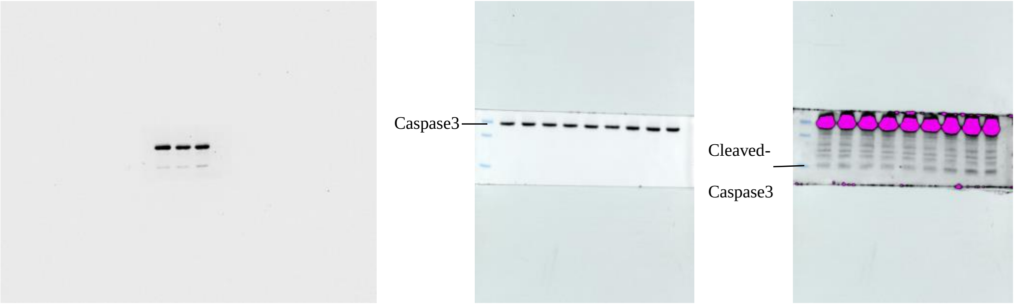

Membrane 1 **Membrane 2** **Membrane 3**

Figure 4 D ATF3 (Membrane1, From left to right: WT 1, Vector 1, Tmprss6 1. Membrane2, From left to right: WT 2, WT 3, WT 4, Vector 2, Vector 3, Vector 4, Tmprss6 2, Tmprss6 3, Tmprss6 4.)

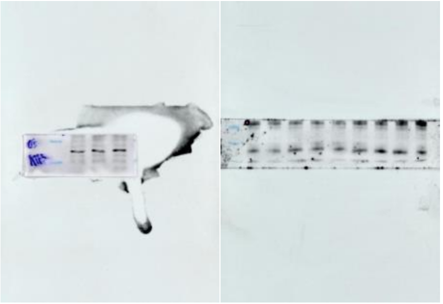

Membrane1 Membrane2

Figure 4 D β-actin to ATF3 (Membrane1, From left to right: WT 1, Vector 1, Tmprss6 1. Membrane2, From left to right: WT 2, WT 3, WT 4, Vector 2, Vector 3, Vector 4, Tmprss6 2, Tmprss6 3, Tmprss6 4.)

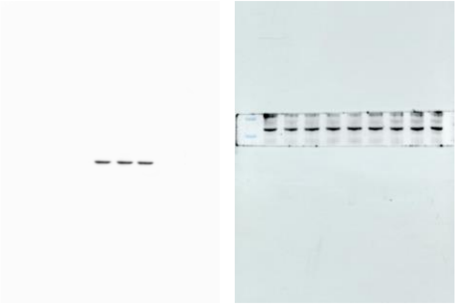

Membrane1 Membrane2

Figure 4I

P-p38

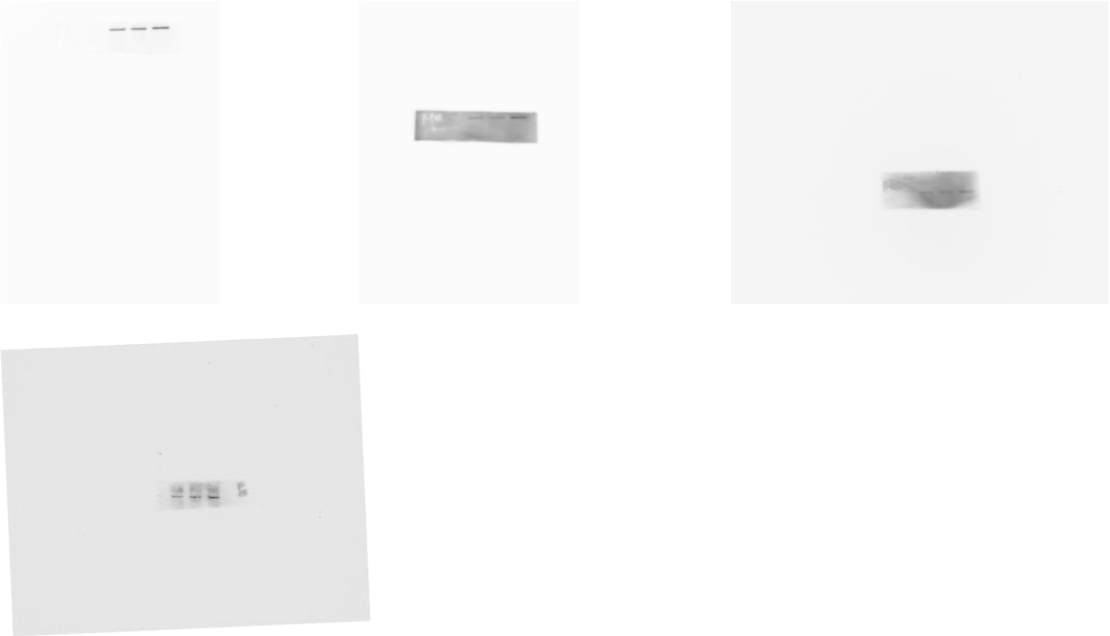

P38

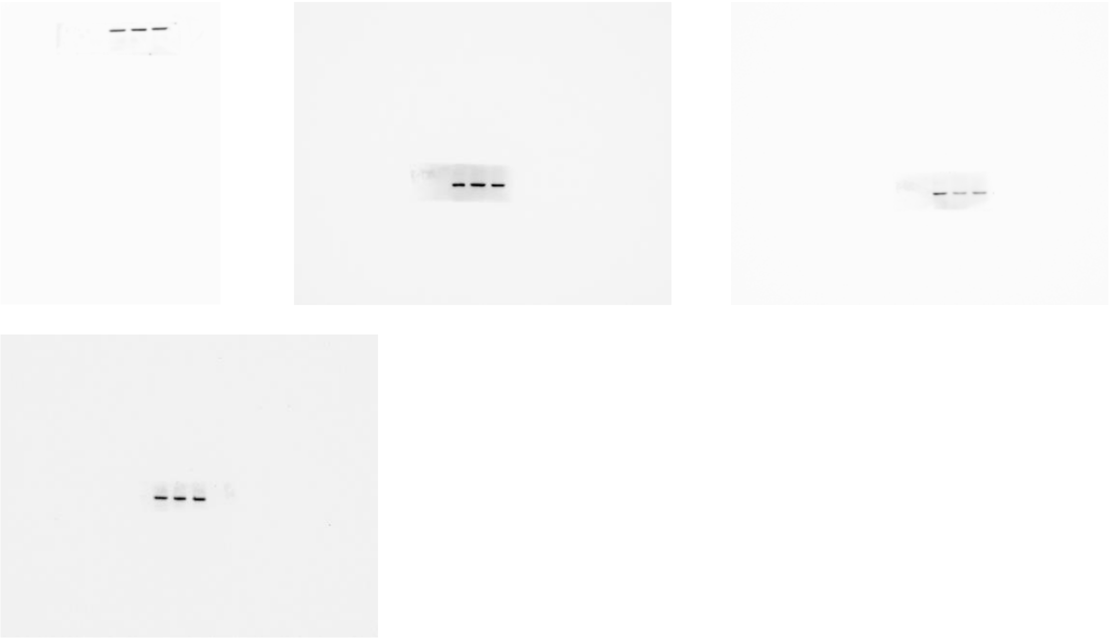

Figure 5 A Tmprss6

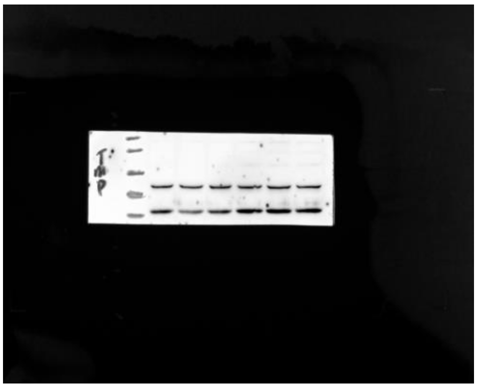

Figure 5 A β-actin to Tmprss6

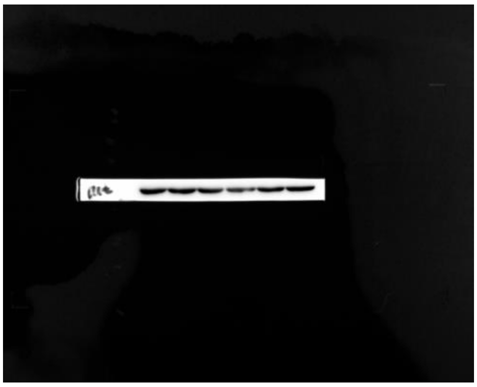

Figure 5 C HJV

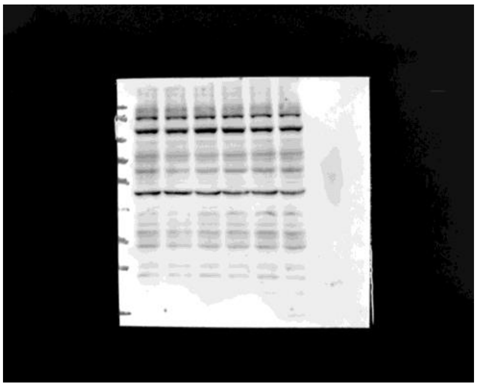

Figure 5 C β-actin to HJV

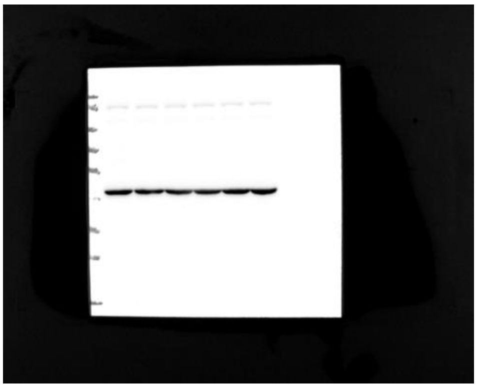

Figure 5 E P-Smad1/5/8

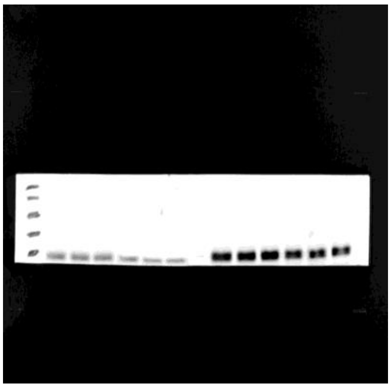

Figure 5 E Smad1

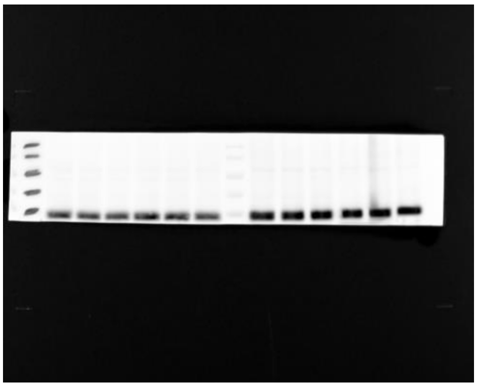

Figure 5 E Smad4

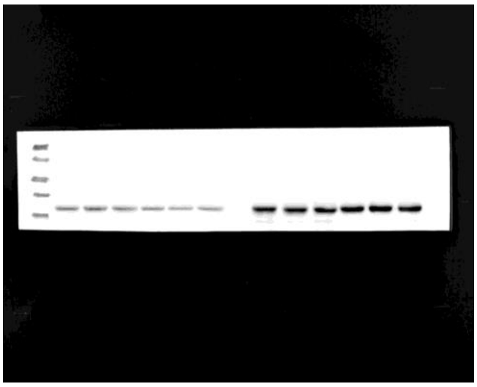

Figure 5 E β-actin to P-Smad1/5/8

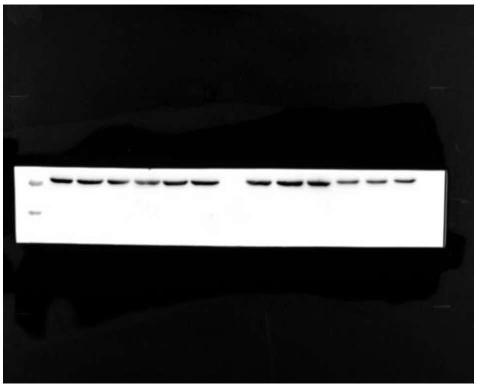

Figure 5 E Histone3 to P-Smad1/5/8

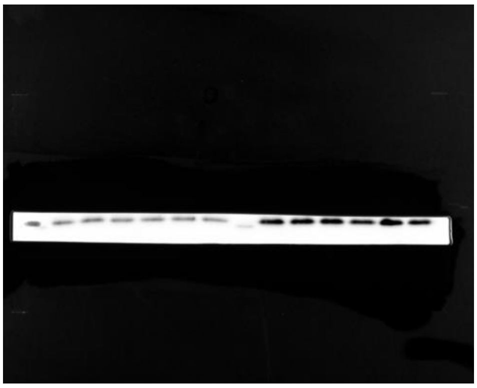

Figure 5 E ATF3

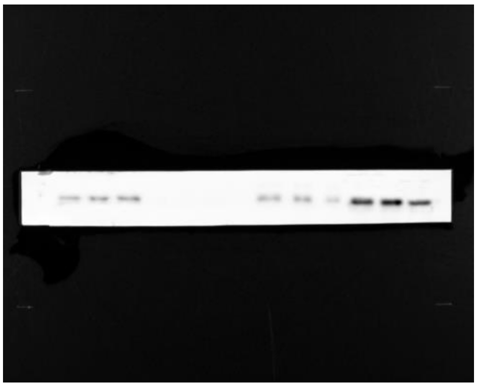

Figure 5 E β-actin to ATF3

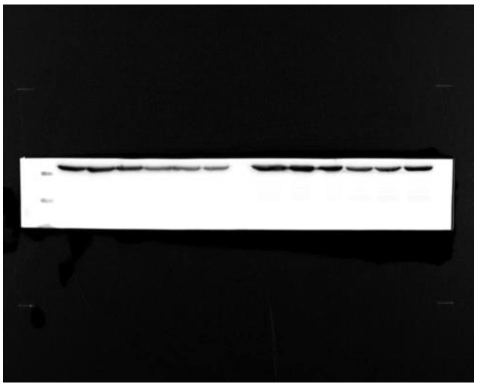

Figure 5 E Histone3 to ATF3

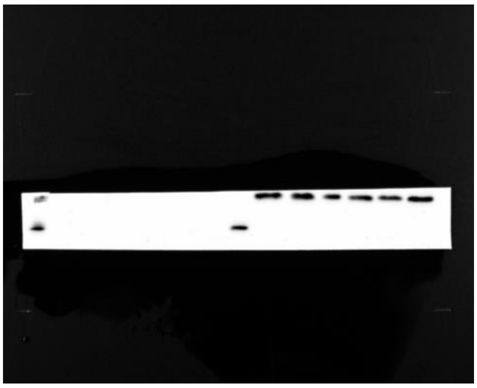

Figure 5 I Pro-hepcidin

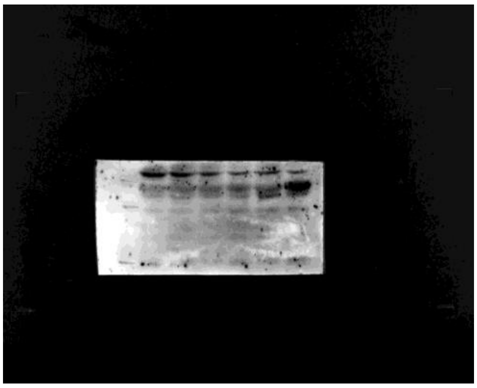

Figure 5 I FPN1

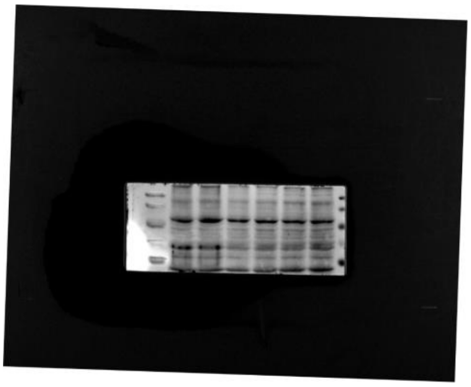

Figure 5 I β-actin to Pro-hepcidin

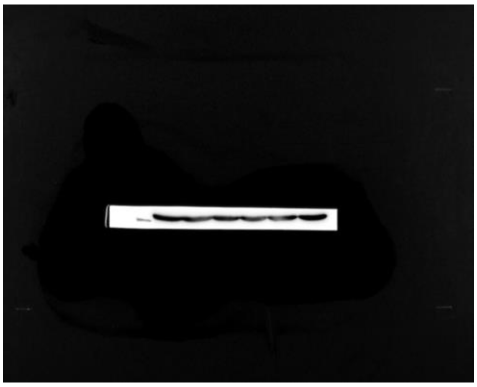

Figure 5 K TfR1

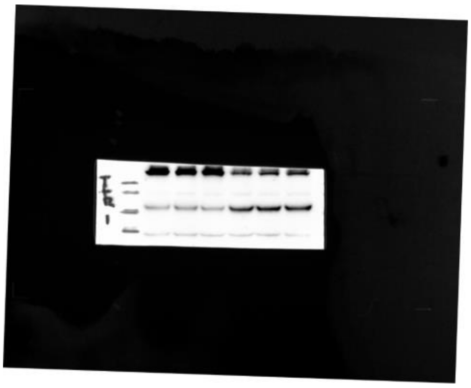

Figure 5 K β-actin to TfR1

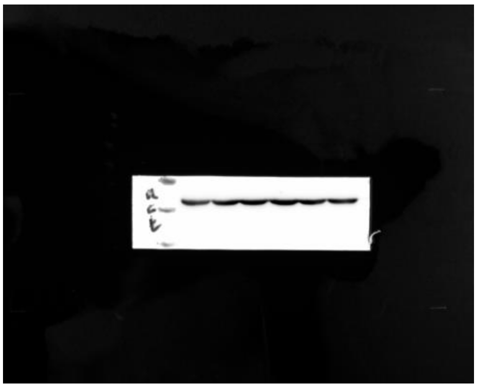

Figure 5 K FtL

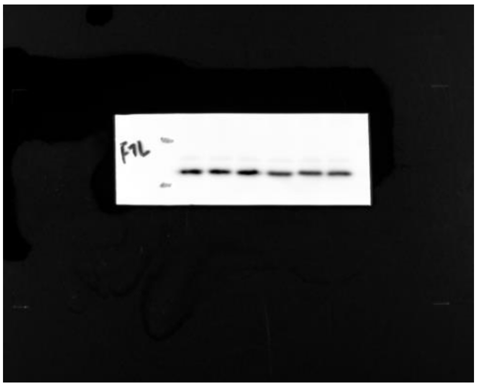

Figure 5 K β-actin to FtL

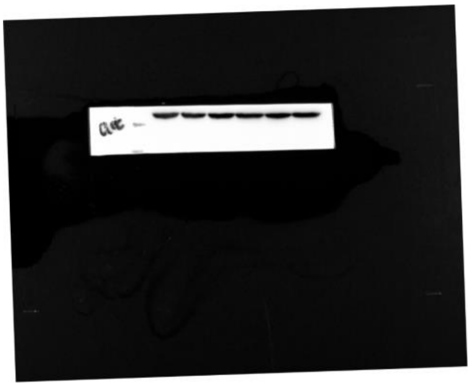

Figure 5 K FtH

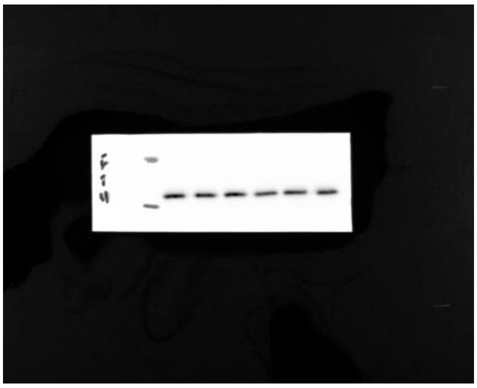

Figure 5 K β-actin to FtH

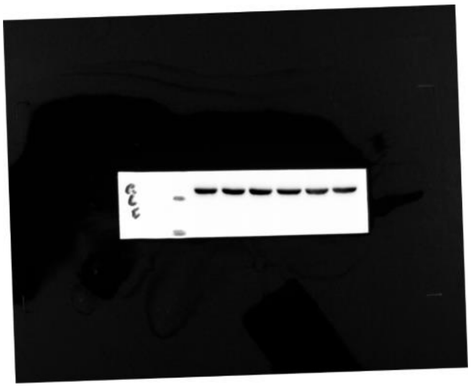

Figure 5 N P-p38

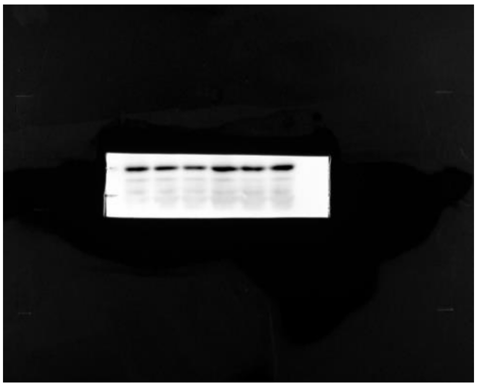

Figure 5 N P38

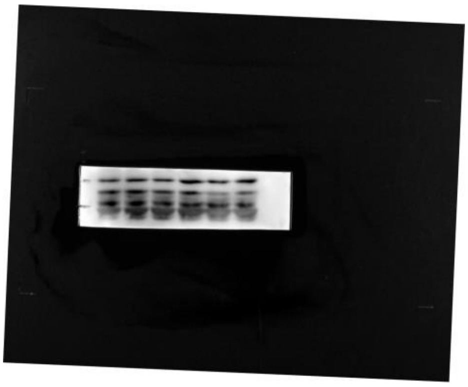

Figure 5 P Bcl-2

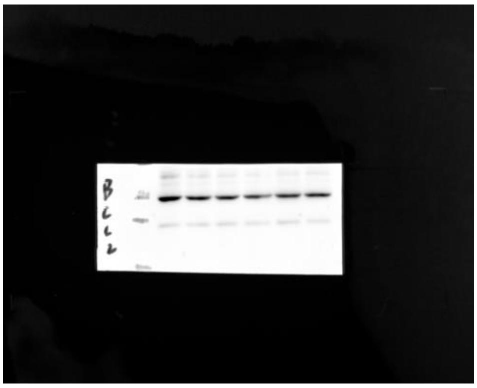

Figure 5 P Bax

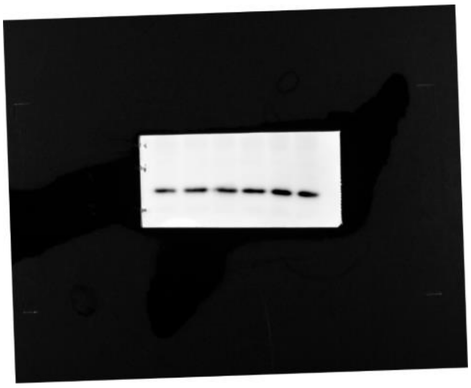

Figure 5 P Caspase3 and Cleaved- Caspase3

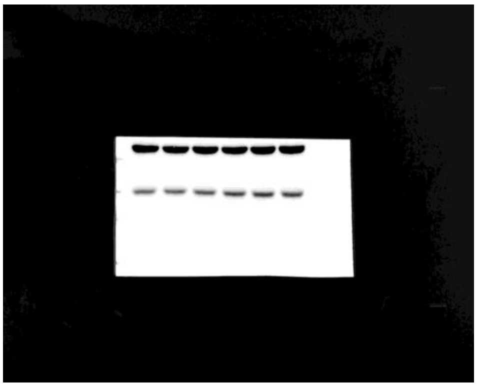

Figure 6 A FLAG (Membrane1, From left to right: WT 1, Vector 1, Tmprss6 1, WT 2, Vector 2, Tmprss6 2. Membrane2, From left to right: WT 3, Vector 3, Tmprss6 3, WT 4, Vector 4, Tmprss6 4.)

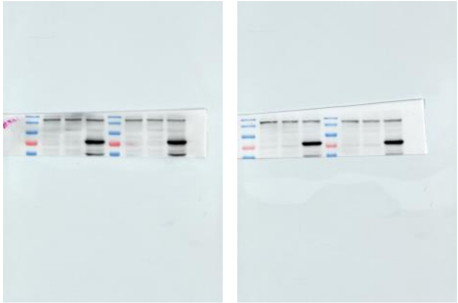

Membrane1 Membrane2

Figure 6 A Smad4

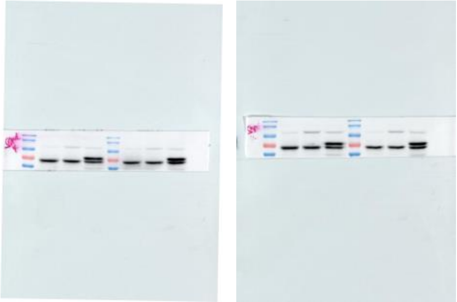

Figure 6 A ATF3

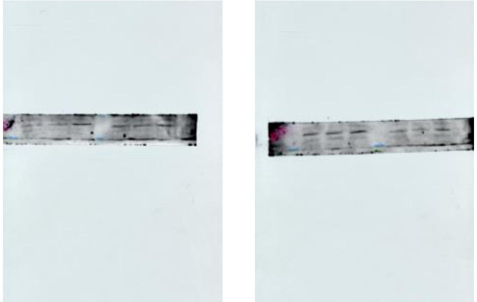

Figure 6 A β-actin to FLAG, Smad4 and ATF3

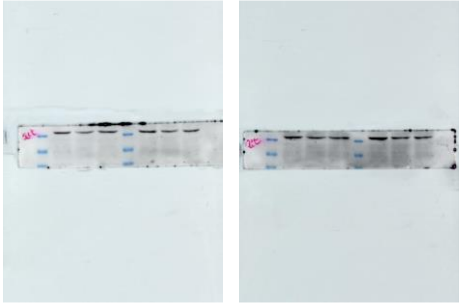

Figure 6 C Smad4

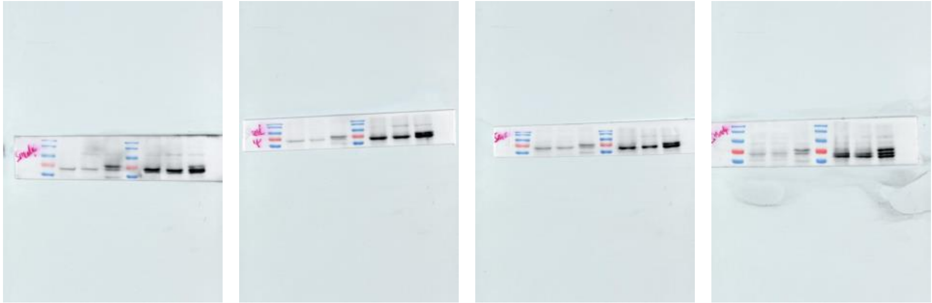

Figure 6 C ATF3

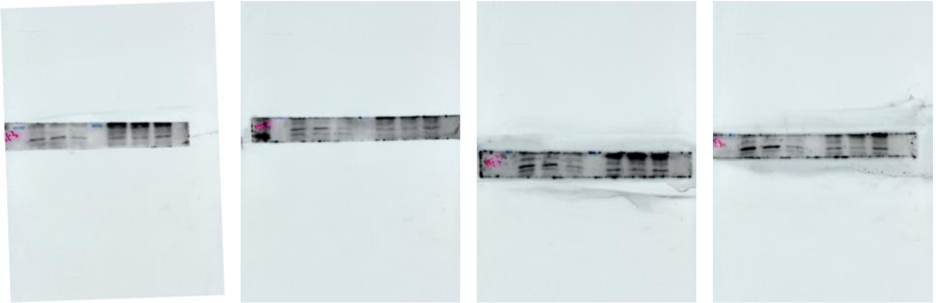

Figure 6 C β-actin to Smad4 and ATF3

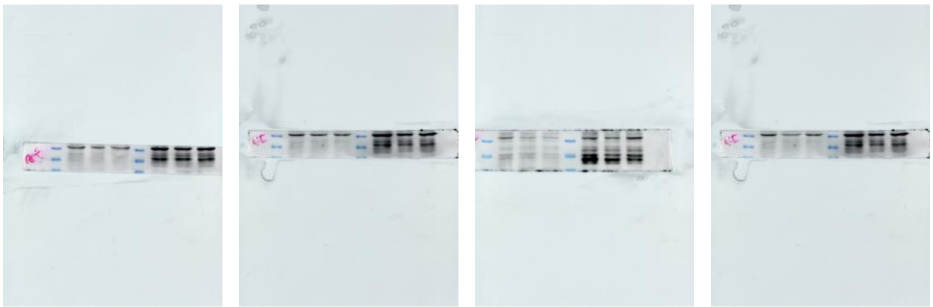

Figure 6 C Histone3 to Smad4 and ATF3

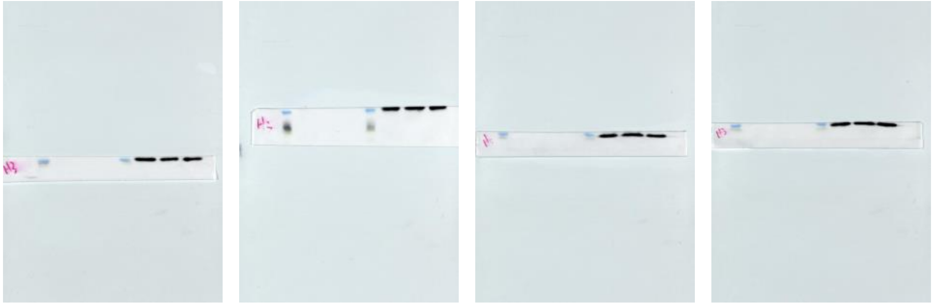

Figure 7 B ATF3

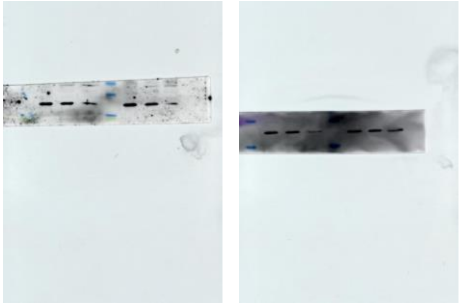

Figure 7 B β-actin to ATF3

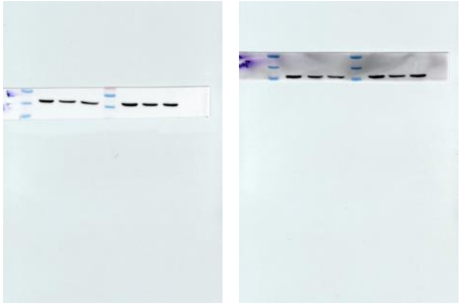

Figure 7 D P-p38

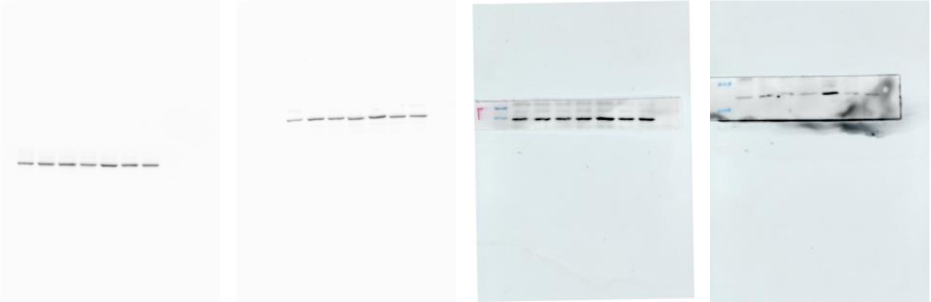

Figure 7 D P38

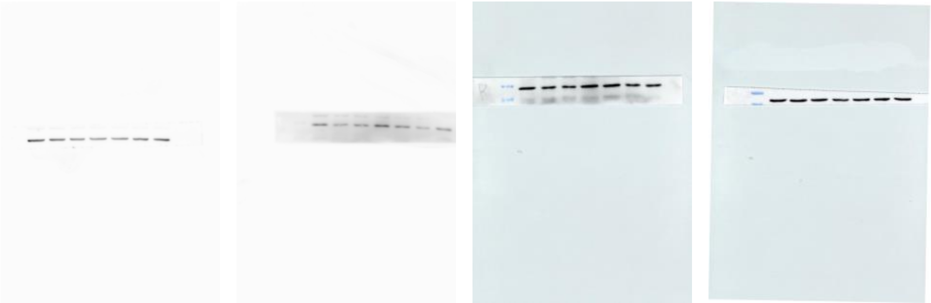

Figure 7 F Bcl-2

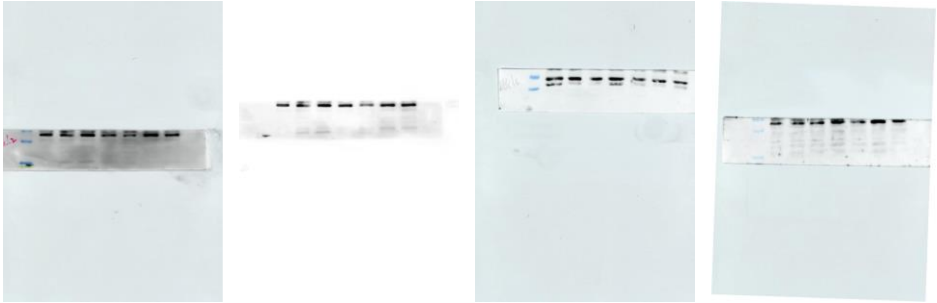

Figure 7 F Bax

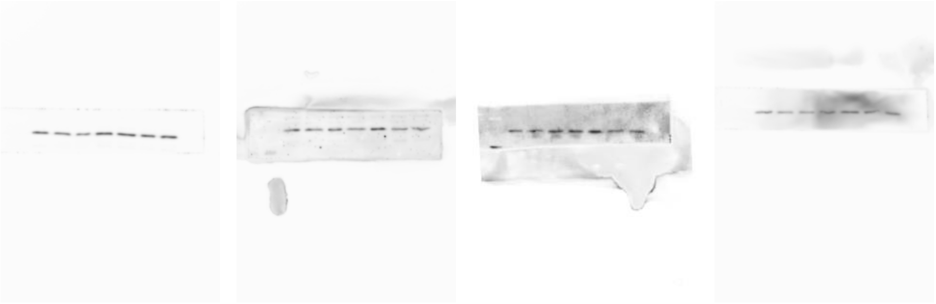

Figure 7 H Caspase3 and Cleaved-Caspase3

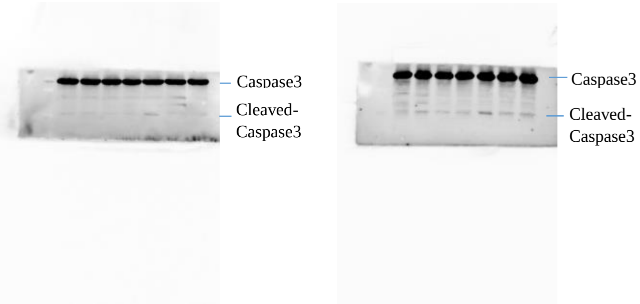

Figure 7 H Caspase3 and Cleaved-Caspase3（Membrane 1 and membrane 2 are the same membrane, Membrane 4 is the image of membrane 3 after overexposure）

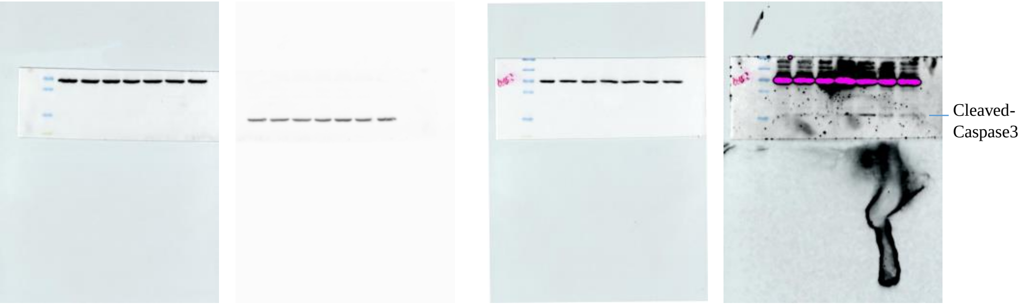

Caspase3（Membrane1） Cleaved-Caspase3（Membrane2） Caspase3（Membrane3） Cleaved-Caspase3（Membrane4）

Figure 8 D FLAG（**Membrane 1**, From left to right: Vector 1, Tmprss6 1, Vector 2, Tmprss6 2, Vector 3, Tmprss6 3. **Membrane 2**, From left to right: Vector 4, Tmprss6 4, Vector 5, Tmprss6 5, Vector 6, Tmprss6 6.）

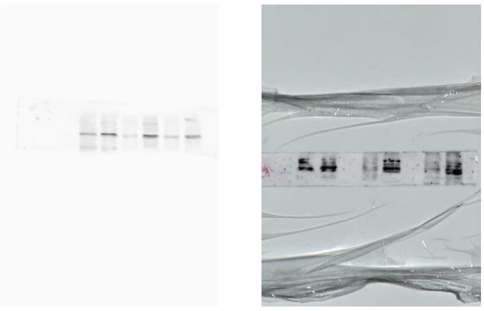

Membrane 1 **Membrane 2**

Figure 8 D β-actin （**Membrane 1**, From left to right: Vector 1, Tmprss6 1, Vector 2, Tmprss6 2, Vector 3, Tmprss6 3. **Membrane 2**, From left to right: Vector 4, Tmprss6 4, Vector 5, Tmprss6 5, Vector 6, Tmprss6 6.）

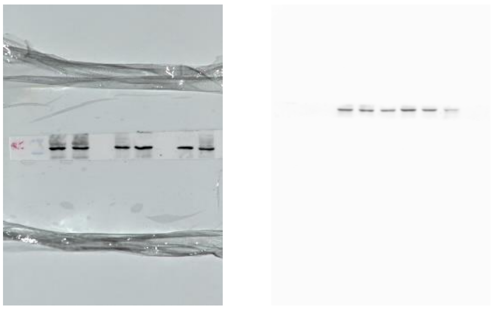

Membrane 1 **Membrane 2**

Figure 8 D Tmprss6 （**Membrane 1**, From left to right: Tmprss6 1, Vector 1, Vector 2, Tmprss6 2, Vector 3, Tmprss6 3. **Membrane 2**, From left to right: Vector 4, Tmprss6 4, Vector 5, Tmprss6 5, Vector 6, Tmprss6 6.）

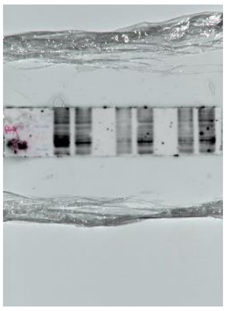 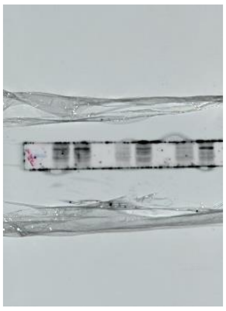

Membrane 1 **Membrane 2**

Figure 8 D β-actin （**Membrane 1**, From left to right: Vector 1, Tmprss6 1, Vector 2, Tmprss6 2, Vector 3, Tmprss6 3. **Membrane 2**, From left to right: Vector 4, Tmprss6 4, Vector 5, Tmprss6 5, Vector 6, Tmprss6 6.）

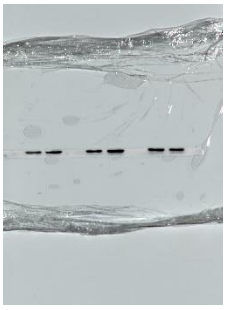 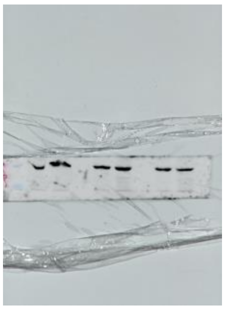

Membrane 1 **Membrane 2**

Figure 8 F ATF3 （**Membrane 1**, From left to right: Vector 1, Tmprss6 1, Vector 2, Tmprss6 2, Vector 3, Tmprss6 3. **Membrane 2**, From left to right: Vector 4, Tmprss6 4, Vector 5, Tmprss6 5, Vector 6, Tmprss6 6.）

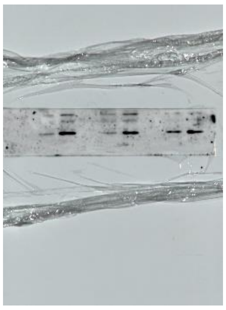 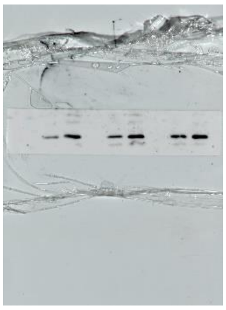

Membrane 1 **Membrane 2**

Figure 8 F β-actin （**Membrane 1**, From left to right: Vector 1, Tmprss6 1, Vector 2, Tmprss6 2, Vector 3, Tmprss6 3. **Membrane 2**, From left to right: Vector 4, Tmprss6 4, Vector 5, Tmprss6 5, Vector 6, Tmprss6 6.）

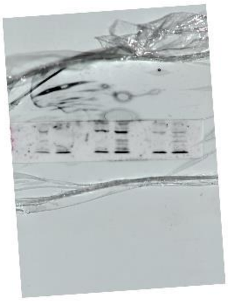 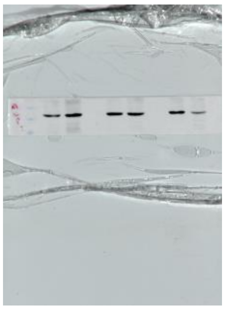

Membrane 1 **Membrane 2**

Figure 8 F P-p38（**Membrane 1**, From left to right: Vector 1, Tmprss6 1, Vector 2, Tmprss6 2, Vector 3, Tmprss6 3. **Membrane 2**, From left to right: Vector 4, Tmprss6 4, Vector 5, Tmprss6 5, Vector 6, Tmprss6 6.）

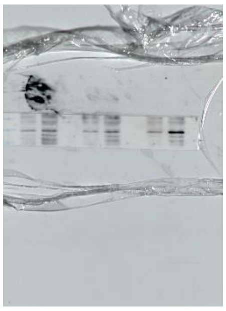 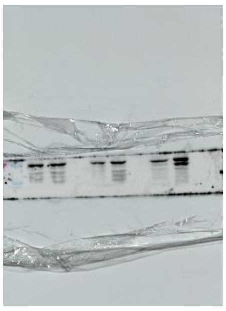

Membrane 1 **Membrane 2**

Figure 8 F P38（**Membrane 1**, From left to right: Vector 1, Tmprss6 1, Vector 2, Tmprss6 2, Vector 3, Tmprss6 3. **Membrane 2**, From left to right: Vector 4, Tmprss6 4, Vector 5, Tmprss6 5, Vector 6, Tmprss6 6.）

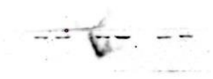 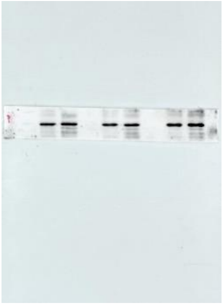

Membrane 1 **Membrane 2**

Figure 8 I Bcl-2（**Membrane 1**, From left to right: Vector 1, Vector 2, Tmprss6 1. **Membrane 2**, From left to right: Vector 3, Tmprss6 2, Vector 4, Tmprss6 3,Vector 5, Tmprss6 4.）

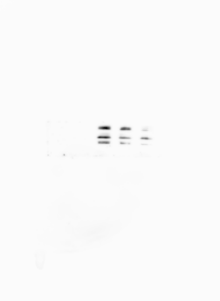
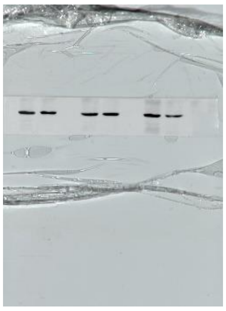

Membrane 1 **Membrane 2**

Figure 8 I Bax （**Membrane 1**, From left to right: Vector 1, Tmprss6 1, Vector 2, Tmprss6 2, Vector 3, Tmprss6 3. **Membrane 2**， From left to right: Vector 4, Vector 5， Tmprss6 4.）

Membrane 1 **Membrane 2**

Figure 8 I β-actin（**Membrane 1**, From left to right: Vector 1, Vector 2, Tmprss6 1. **Membrane 2**, From left to right: Vector 3, Tmprss6 2, Vector 4, Tmprss6 3,Vector 5, Tmprss6 4.）

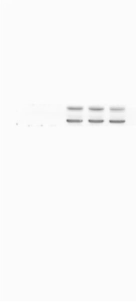
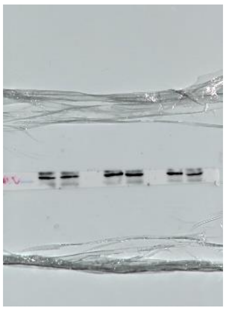

Membrane 1 **Membrane 2**

Figure 8 K Caspase3 and Cleaved-caspase3 （**Membrane 1**, From left to right: Vector 1, Tmprss6 1, Vector 2, Tmprss6 2, Vector 3, Tmprss6 3. **Membrane 2**, From left to right: Vector 4, Tmprss6 4, Vector 5, Tmprss6 5.）

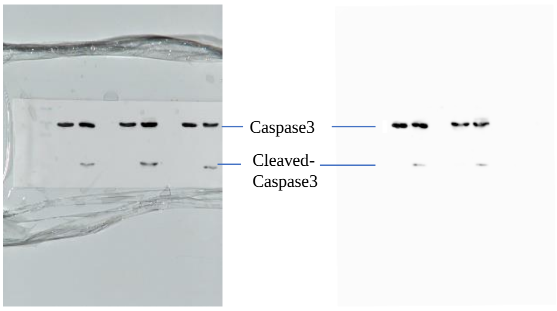

Membrane 1 **Membrane 2**

Figure S2 A ACSL4 (Membrane1, From left to right: WT 1, Vector 1, Tmprss6 1. Membrane2, From left to right: WT 2, WT 3, WT 4, Vector 2, Vector 3, Vector 4, Tmprss6 2, Tmprss6 3, Tmprss6 4.)

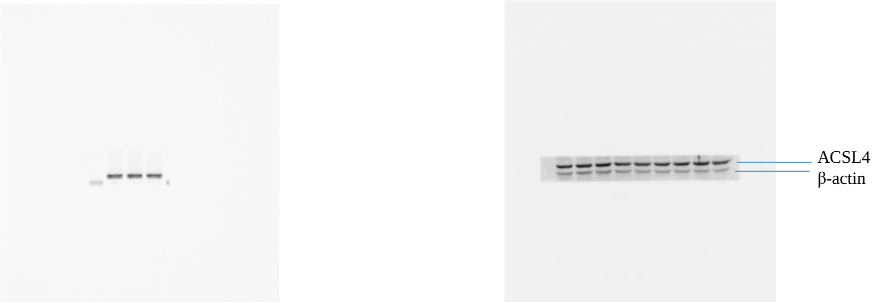

Membrane1 Membrane2

Figure S2 A GPX4 (Membrane1, From left to right: WT 1, Vector 1, Tmprss6 1. Membrane 2, From left to right: WT 2, WT 3, WT 4, Vector 2, Vector 3, Vector 4, Tmprss6 2, Tmprss6 3, Tmprss6 4.)

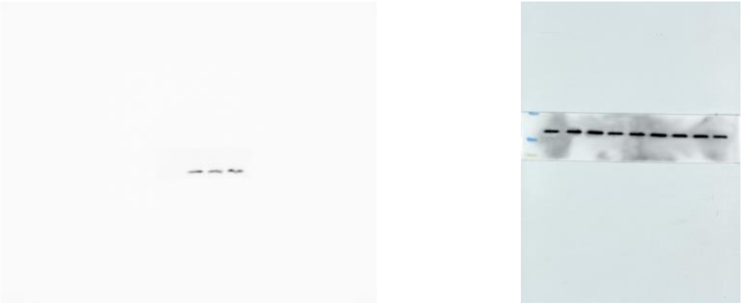

Membrane1 Membrane2

Figure S2 A (Membrane1, From left to right: WT 1, Vector 1, Tmprss6 1. Membrane 2, From left to right: WT 2, WT 3, WT 4, Vector 2, Vector 3, Vector 4, Tmprss6 2, Tmprss6 3, Tmprss6 4.)

β-actin to ACSL4 and GPX4 β-actin to GPX4

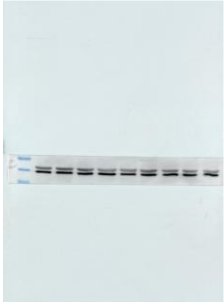

Membrane1 Membrane2

Figure S2 C RIP1 (Membrane1, From left to right: WT 1, Vector 1, Tmprss6 1. Membrane2, From left to right: WT 2, WT 3, WT 4, Vector 2, Vector 3, Vector 4, Tmprss6 2, Tmprss6 3, Tmprss6 4.)

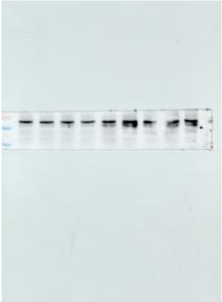

Membrane1 Membrane2

Figure S2 C RIP3 (Membrane1, From left to right: WT 1, Vector 1, Tmprss6 1. Membrane2, From left to right: WT 2, WT 3, WT 4, Vector 2, Vector 3, Vector 4, Tmprss6 2, Tmprss6 3, Tmprss6 4.)

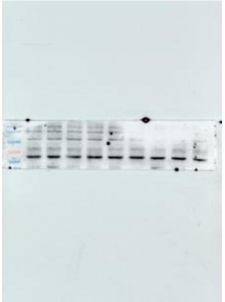

Membrane1 Membrane2

Figure S2 C β-actin (Membrane1, From left to right: WT 1, Vector 1, Tmprss6 1. Membrane2, From left to right: WT 2, WT 3, WT 4, Vector 2, Vector 3, Vector 4, Tmprss6 2, Tmprss6 3, Tmprss6 4. Membrane3, From left to right: WT 2, WT 3, WT 4, Vector 2, Vector 3, Vector 4, Tmprss6 2, Tmprss6 3, Tmprss6 4.)

β-actin to RIP1 and RIP3

Membrane1

β-actin to RIP1

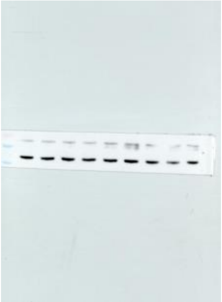

Membrane2

β-actin to RIP3

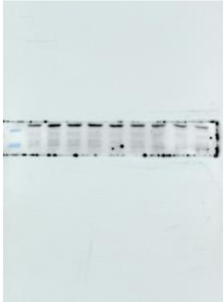

Membrane3

Figure S4 A Tmprss6

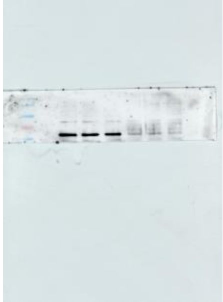

Figure S4 A β-actin to Tmprss6

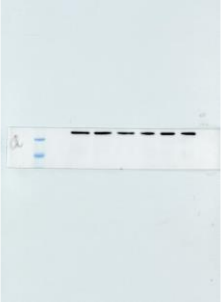

Figure S4 C P-Smad1/5/8

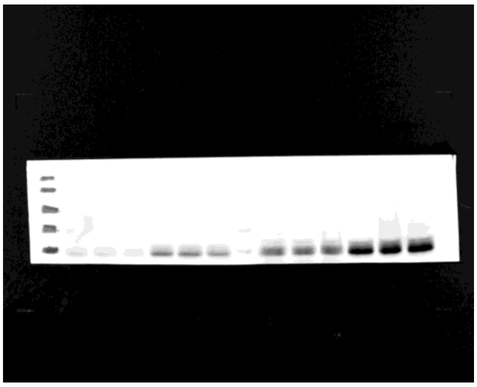

Figure S4 C Smad1

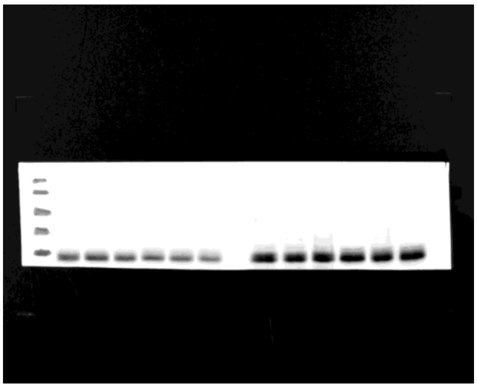

Figure S4 C Smad4

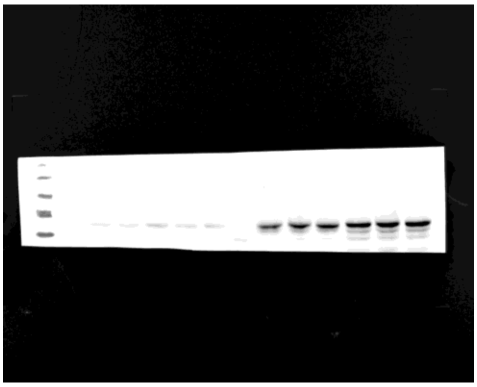

Figure S4 C β-actin to Smad4

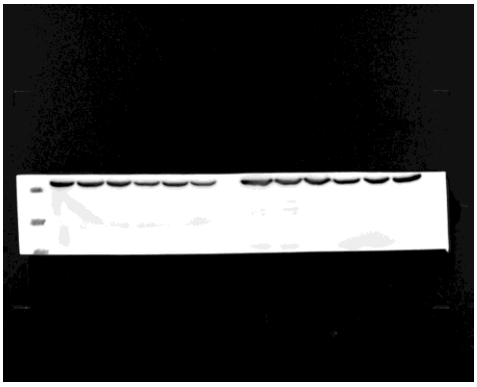

Figure S4 C Histone 3 to Smad4

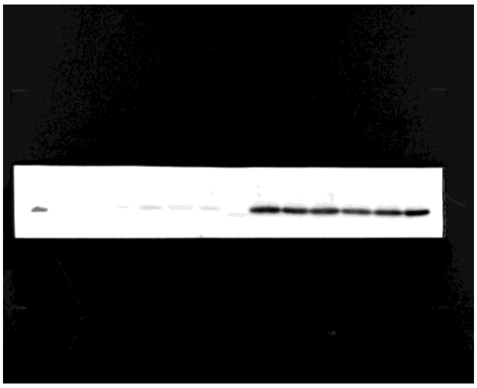

Figure S4 C ATF3

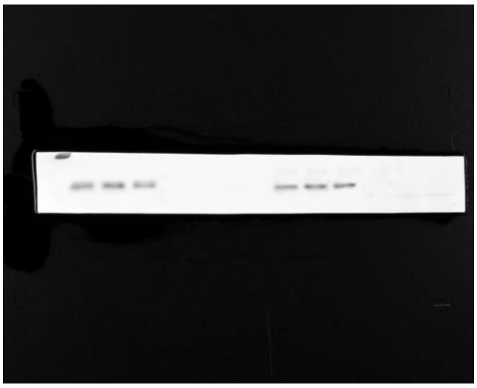

Figure S4 C β-actin to ATF3

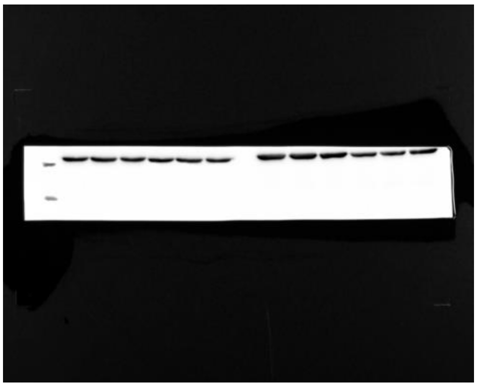

Figure S4 C Histone 3 to ATF3

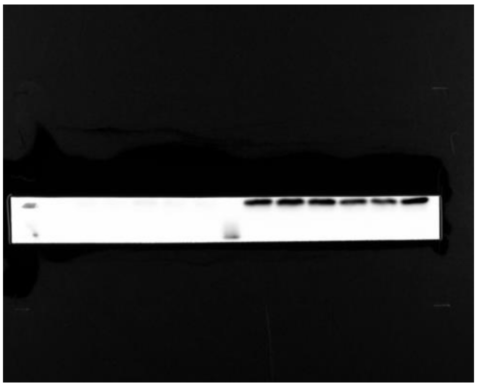

Supplement: Supplementary file 2 — Original Data File [file 41419_2024_6442_MOESM2_ESM.pdf]
